# Novel thiazolidinedione analog reduces a negative impact on bone and mesenchymal stem cell properties in obese mice compared to classical thiazolidinediones

**DOI:** 10.1016/j.molmet.2022.101598

**Published:** 2022-09-11

**Authors:** Andrea Benova, Michaela Ferencakova, Kristina Bardova, Jiri Funda, Jan Prochazka, Frantisek Spoutil, Tomas Cajka, Martina Dzubanova, Tim Balcaen, Greet Kerckhofs, Wouter Willekens, G. Harry van Lenthe, Glenda Alquicer, Alena Pecinova, Tomas Mracek, Olga Horakova, Martin Rossmeisl, Jan Kopecky, Michaela Tencerova

**Affiliations:** 1 Laboratory of Molecular Physiology of Bone, Institute of Physiology of the Czech Academy of Sciences, Prague 142 20, Czech Republic; 2 Faculty of Science, Charles University, Prague, Czech Republic; 3 Laboratory of Adipose Tissue Biology, Institute of Physiology of the Czech Academy of Sciences, Prague 142 20, Czech Republic; 4 Czech Centre for Phenogenomics & Laboratory of Transgenic Models of Diseases, Institute of Molecular Genetics of the Czech Academy of Sciences, Prague, Czech Republic; 5 Laboratory of Translational Metabolism, Institute of Physiology of the Czech Academy of Sciences, Prague 142 20, Czech Republic; 6 Biomechanics lab, Institute of Mechanics, Materials, and Civil Engineering, UCLouvain, Louvain-la-Neuve, Belgium; 7 Department of Materials Engineering, KU Leuven, Belgium; 8 Prometheus, Division of Skeletal Tissue Engineering, Katholieke Universiteit Leuven, 3000 Leuven, Belgium; 9 Pole of Morphology, Institute for Experimental and Clinical Research, UCLouvain, Brussels, Belgium; 10 Department of Chemistry, Molecular Design and Synthesis, KU Leuven, Leuven, Belgium; 11 FIBEr, KU Leuven, Leuven, Belgium; 12 Department of Mechanical Engineering, KU Leuven, Leuven, Belgium; 13 Laboratory of Bioenergetics, Institute of Physiology of the Czech Academy of Sciences, Prague, Czech Republic

**Keywords:** Obesity-induced bone fragility, Bone microstructure, Bone marrow mesenchymal stem cells, Bone marrow adiposity, Thiazolidinedione analog MSDC-0602K, Pioglitazone

## Abstract

**Objective:**

The use of thiazolidinediones (TZDs) as insulin sensitizers has been shown to have side effects including increased accumulation of bone marrow adipocytes (BMAds) associated with a higher fracture risk and bone loss. A novel TZD analog MSDC-0602K with low affinity to PPARγ has been developed to reduce adverse effects of TZD therapy. However, the effect of MSDC-0602K on bone phenotype and bone marrow mesenchymal stem cells (BM-MSCs) in relation to obesity has not been intensively studied yet.

**Methods:**

Here, we investigated whether 8-week treatment with MSDC-0602K has a less detrimental effect on bone loss and BM-MSC properties in obese mice in comparison to first generation of TZDs, pioglitazone. Bone parameters (bone microstructure, bone marrow adiposity, bone strength) were examined by μCT and 3-point bending test. Primary BM-MSCs were isolated and measured for osteoblast and adipocyte differentiation. Cellular senescence, bioenergetic profiling, nutrient consumption and insulin signaling were also determined.

**Results:**

The findings demonstrate that MSDC-0602K improved bone parameters along with increased proportion of smaller BMAds in tibia of obese mice when compared to pioglitazone. Further, primary BM-MSCs isolated from treated mice and human BM-MSCs revealed decreased adipocyte and higher osteoblast differentiation accompanied with less inflammatory and senescent phenotype induced by MSDC-0602K vs. pioglitazone. These changes were further reflected by increased glycolytic activity differently affecting glutamine and glucose cellular metabolism in MSDC-0602K-treated cells compared to pioglitazone, associated with higher osteogenesis.

**Conclusion:**

Our study provides novel insights into the action of MSDC-0602K in obese mice, characterized by the absence of detrimental effects on bone quality and BM-MSC metabolism when compared to classical TZDs and thus suggesting a potential therapeutical use of MSDC-0602K in both metabolic and bone diseases.

## Highlights

• MSDC-0602K improves bone quality and increases proportion of smaller BMAds in obese mice.
• MSDC-0602K-treated mice show lower adipogenic differentiation with less senescent phenotype in primary BM-MSCs.
• MSDC-0602K induces higher glycolytic activity in BM-MSCs compared to pioglitazone.
• MSDC-0602-treated BM-MSCs prefer glutamine over glucose uptake in comparison to AT-MSCs.
• Beneficial effect of MSDC-06002K in BM-MSCs manifests by absence of MPC inhibition.

## Graphical abstract

**Figure:**
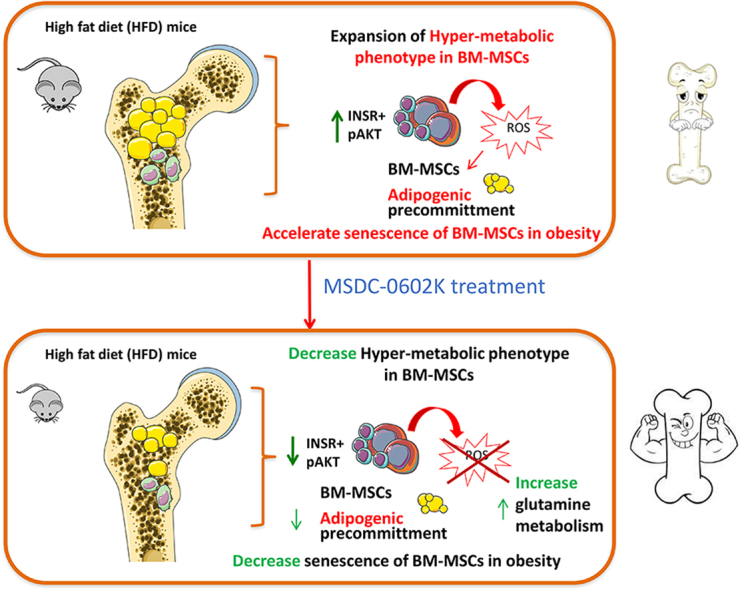

## Abbreviations

*ALP*: Alkaline phosphatase
*AT*: Adipose tissue
*BM*: Bone marrow
*BMAds*: Bone marrow adipocytes
*BMAT*: Bone marrow adipose tissue
*BM-MSCs*: Bone marrow mesenchymal stem cells
*CE-CT*: Contrast-enhanced microCT
*CESA*: Contrast-enhancing staining agent
*ECAR*: Extracellular acidification rate
*HFD*: High-fat diet
*HSCs*: Hematopoietic stem cells
*GTT*: Glucose tolerance test
*LPC*: Lysophophatidylcholine
*MPC*: mitochondrial pyruvate carrier
*ND*: Normal diet
*OCR*: Oxygen consumption rate
*P1NP*: Procollagen type 1 N-terminal propeptide
*POM*: Polyoxometalate
*ROS*: Reactive oxygen species
*TG*: Triacylglycerol
*TRAP*: Tartrate-resistant acid phosphatase
*TZDs*: Thiazolidinediones

## 1 Introduction

Obesity is accompanied with ectopic fat accumulation in non-adipose organs, including bones, leading to increased bone marrow (BM) adiposity, which is associated with an increased risk of bone fractures and osteoporosis in obese and diabetic patients [1]. Several studies including our recent observations, show that obesity changes the BM microenvironment and impacts BM mesenchymal stem cell (BM-MSC) properties by increased adipogenesis, which contributes to bone fragility induced by metabolic complications [[2], [3], [4], [5], [6]].

In obesity and type 2 diabetes several approaches are used to treat or prevent the detrimental effects of metabolic diseases, including physical activity, dietary or pharmacological treatment. The use of thiazolidinediones (TZDs) has been for a long time considered as an appropriate treatment for metabolic complications by improving insulin sensitivity in patients with type 2 diabetes. However, the adverse effects induced by first generation of TZDs in terms of weight gain, cardiovascular complications and bone loss have reduced their use in the clinical practice [7]. The negative effect of TZDs on bone physiology is associated with increased BM adiposity leading to bone loss [7]. The use of these insulin-sensitizing drugs usually has a secondary detrimental effect on bone physiology because of the activation of peroxisome proliferator-activated receptor-γ (PPARγ) regulating adipocyte (AD) differentiation both in the periphery and in the BM, where they also induce osteoclast activation [5,8]. Therefore, further studies are needed to dissect the molecular effects of this type of antidiabetic treatments on bone and fat physiology.

A previous study by Stechschulte et al. [9] demonstrated that changes in post-translational modification of PPARγ can reduce the negative effect on bone metabolism while maintaining the positive effect on energy metabolism. Along these lines, a novel “PPARγ-sparing” TZD analog MSDC-0602K (“second generation of TZDs”) was developed to minimize the adverse side effects of TZDs on bone and fat metabolism [[10], [11], [12], [13], [14]]. MSDC-0602K was purposely designed to decrease direct binding to PPARγ [11,15], but it maintains its inhibitory effect on mitochondrial pyruvate carrier (MPC), which likely contributes to the beneficial effect on energy metabolism and glucose uptake [11,12,16]. Testing of MSDC-0602K drug in a clinical trial (phase 2b) showed promising results in terms of lowering glucose and insulin levels and improving liver steatosis without side effects in obese subjects [17]. Fukunaga et al. [13] recently reported that MSDC-0602K showed no side effects in lean mice with respect to bone parameters compared to rosiglitazone. However, the impact of this novel TZD analog on bone metabolism in obese conditions has not been studied yet. Thus, the main objective of this study was to determine whether MSDC-0602K has a less detrimental effect on bone structure and molecular properties of BM-MSCs compared to a typical TZD pioglitazone, using a model of diet-induced obesity in male C57BL/6N mice.

## 2 Materials and methods

Additional methods are described in the Supplementary Material.

### 2.1 Animals and dietary interventions

Male C57BL/6N mice (Charles River Laboratories, Sulzfeld, Germany) were maintained at 22 °C with 12-hour light–dark cycle (light from 6:00 a.m.). Upon arrival and before the start of the experiment all mice had free access to water and standard chow diet (ND, Ssniff Spezialdieten GmbH, Soest, Germany). At the age of 12 weeks, mice were randomly divided into 5 groups (n = 8–10, repeated in 3 independent experiments) and fed for 8 weeks with: i) normal diet (ND) (3.4% wt/wt as lipids; Rat/Mouse- Maintenance extrudate; Ssniff Spezialdieten GmbH, Soest, Germany); ii) high-fat diet (HFD, lipid content, ∼35% wt/wt, mainly corn oil [18]; iii) HFD + P, HFD supplemented with 50 mg pioglitazone/kg diet (Actos, Takeda, Japan); iv) HFD + M, HFD supplemented with 330 mg MSDC-0602K/kg diet (Cirius Therapeutics, USA) [10]. Diets were stored at −20 °C, in sealed plastic bags filled with nitrogen. The dose of pioglitazone and MSDC-0602K was the same as in the previous studies using these compounds in mice [10,18]. Body weight and 24-hour food consumption were measured every week.

Mice were sacrificed after 8 weeks of dietary interventions in the fed-state by cervical dislocation under diethyl ether anesthesia. Tissue samples and primary isolated mBM-MSCs were used for subsequent molecular analyses. All experiments were performed according to the guideline of the Institute of Physiology of the Czech Academy of Sciences and were approved under protocol number 81/2016.

#### 2.1.1 Glucose tolerance test

Intraperitoneal glucose tolerance test (GTT) was performed using 1 mg of glucose/g body weight in overnight fasted mice as previously published [10].

#### 2.1.2 Biochemical analysis of plasma

Blood glucose levels were measured by OneTouch Ultra glucometers (LifeScan, Milpitas, CA, USA), and plasma insulin levels were determined by the Sensitive Rat Insulin RIA Kit (Millipore, Billerica, MA, USA).

### 2.2 Isolation of mBM-MSCs and mHSCs

mBM-MSCs were isolated from the bones of front and hind limbs of C57BL/6N male mice (after 8 weeks of dietary treatments) following previous protocols with the small modifications [3]. After bone crushing, collagenase digestion (StemCell, Vancouver, BC, Canada) and negative selection of CD45, CD31 and Ter119 cells (Miltenyi, Bergisch Gladbach, Germany) mBM-MSCs were obtained. mBM-MSCs were subcultured in growth medium (MEM alpha (Thermo Fisher Scientific, Waltham, MA, USA) + 20% FBS (Thermo Fisher Scientific, Waltham, MA, USA) + 1% penicillin/streptomycin (P/S) (Thermo Fisher Scientific, Waltham, MA, USA) + 0.5% Amphotericin B (Merck, Darmstadt, Germany) + 1% Glutamax (Thermo Fisher Scientific, Waltham, MA, USA) + 1% MEM NEAA (Thermo Fisher Scientific, Waltham, MA, USA) + 1% sodium pyruvate (Thermo Fisher Scientific, Waltham, MA, USA)) and cultivated for further analysis. The positive fraction with mHSCs was divided into three samples. One was harvested for mRNA isolation with Tri-Reagent (Merck, Darmstadt, Germany), the second was harvested for proteins and the rest was seeded and cultivated in growth medium for further analysis.

#### 2.2.1 Colony forming units-fibroblast (CFU-f) assay

After mBM-MSC isolation, cells were seeded for CFU 500 cells/60 mm Petri dish and cultivated in growth media. After 14 days in culture colonies displaying more than 50 cells were counted using Crystal Violet staining (Merck, Darmstadt, Germany).

#### 2.2.2 Short-time proliferation assay

Isolated mBM-MSCs were plated in 24-well plate in triplicates at density of 1000 cells/well in standard growth medium. Cell number was evaluated after 1, 3, 6 and 9 days. Cells were washed with PBS, detached by trypsinization, and then manually counted using Bürker-Türk counting chamber.

#### 2.2.3 *In vitro* differentiation of mBM-MSCs

Primary mBM-MSCs from passage 2 were used for analyzing their differentiation capacity.

##### 2.2.3.1 Osteoblast differentiation

mBM-MSCs were seeded at a density of 20 000 cells/cm^2^. When the cells reached 80% confluence MEM medium (Thermo Fisher Scientific, Waltham, MA, USA) supplemented with 10% FBS (Thermo Fisher Scientific, Waltham, MA, USA) and 1% P/S (Thermo Fisher Scientific, Waltham, MA, USA) was added to control cells and the rest of the cells were cultivated with osteoblast induction media consisting of 10 mM β-glycerophosphate (Merck, Darmstadt, Germany), 10 nM dexamethasone (Merck, Darmstadt, Germany) and 50 μg/ml Vitamin C (Wako Chemicals USA Inc., Richmond, VA, USA). The media was changed every second day for 7 days (ALP activity) and 11 days (Alizarin Red staining).

##### 2.2.3.2 Alizarin Red staining

Mineralization of cell matrix at day 11 was measured using Alizarin Red S staining. Cells were fixed with 70% ice-cold ethanol for minimum 1 h at −20 °C after which Alizarin Red S solution (Merck, Darmstadt, Germany) was added. The cells were stained for 10 min at room temperature (RT). Excess dye was washed with distilled water followed by PBS. The amount of mineralized matrix (bound stain) was quantified by elution of the Alizarin red stain, using 20 min incubation of the cultures in 70% dH2O, 20% ethanol and 10% methanol solution on a shaker (100 rpm) at RT. The absorbance of the eluted dye was measured at 500 nm, using microplate reader according to the protocol [19].

##### 2.2.3.3 Alkaline phosphatase (ALP) activity assay

ALP activity and cell viability assay were quantified at day 7 of OB differentiation in order to normalize the ALP activity data to the number of viable cells.

Cell viability assay was performed using Cell Titer-Blue Assay Reagent (Promega, Madison, WI, USA) at fluorescence intensity (579_Ex_/584_Em_). ALP activity was determined by absorbance at 405 nm using p-nitrophenyl phosphate (Merck, Darmstadt, Germany) as substrate [2].

##### 2.2.3.4 Adipocyte differentiation

Cells were plated at density of 30 000 cells/cm^2^. For AD differentiation DMEM media (Thermo Fisher Scientific, Waltham, MA, USA) was used, containing 10% FBS (Thermo Fisher Scientific, Waltham, MA, USA), 9% horse serum (Merck, Darmstadt, Germany), 1% P/S (Thermo Fisher Scientific, Waltham, MA, USA), 100 nM dexamethasone (Merck, Darmstadt, Germany), 0.5 uM 3-isobutyl-1-methyxanthine (IBMX) (Merck, Darmstadt, Germany), 1 μM BRL (Merck, Darmstadt, Germany), 3 μg/mL Insulin (Merck, Darmstadt, Germany). The media was changed every three days for 10 days. Horse serum supplementation of media was used just for the first three days of induction.

##### 2.2.3.5 Oil Red O staining

At day 10 of differentiation cells were rinsed with PBS and fixed in 4% paraformaldehyde (Merck, Darmstadt, Germany) for 10 min at RT. After fixation cells were briefly rinsed with 3% isopropanol solution (Merck, Darmstadt, Germany) and lipid droplets were stained with Oil Red O solution (Merck, Darmstadt, Germany) for 1 h at RT.

##### 2.2.3.6 Nile Red staining

Nile Red Staining was performed as we previously described [20]. It is a direct stain for the detection of intracellular lipid droplets by fluorescence microscopy. Cells were cultured in polystyrene flat-bottom 96-well tissue culture-treated black microplates (BRANDplates®, cellGrade™, Brand, Wertheim, Germany). Nile Red dye (Merck, Darmstadt, Germany) working solution was prepared from a stock solution of 1 mg/ml. Cells were washed with PBS (Thermo Fisher Scientific, Waltham, MA, USA). Dye was added directly to the cells (5 μg/ml in PBS), and incubated for 10 min at room temperature in the dark, then washed twice with PBS. Fluorescent signal was measured using a Cytation 3 cell imaging multimode plate reader (BioTek, Winooski, VT, USA) using excitation of 485 nm and emission of 572 nm. The fluorescent signal of Nile Red stain was normalized to cell viability signal measured by Cell Titer-Blue Assay Reagent (Promega, Madison, WI, USA) mentioned above.

### 2.3 Insulin responsiveness of mHSCs

Primary mHSCs were cultured up to passage 1 and seeded for insulin and LPS stimulation. Cells were plated at a density of 300 000 cells/well in 12-well plates. After reaching the confluence, cells were starved for 4 h in serum-free MEM alpha (Thermo Fisher Scientific, Waltham, MA, USA) medium with 0.5% BSA and 1% P/S, then stimulated with 100 nM Insulin for 15 min at 37 °C and harvested for protein used in subsequent analyses.

### 2.4 *In vitro* differentiation of hBM-MSCs

We used well-characterized hBM-MSC-TERT cell line (as a model of hBM-MSCs) established by ectopic expression of the catalytic subunit of human telomerase, as described previously in our papers [21,22]. Cells were cultured in standard culture medium containing minimal essential medium (MEM) (Thermo Fisher Scientific, Waltham, MA, USA) supplemented with 10% fetal bovine serum (FBS) and 1% penicillin/streptomycin (P/S) (Thermo Fisher Scientific, Waltham, MA, USA) at 37 °C in a humidified atmosphere containing 5% CO_2_. Cells were regularly tested for mycoplasma contamination.

For AD differentiation, cells were plated at a density of 30 000 cells/cm^2^ and induced with adipogenic induction medium DMEM containing 10% FBS (Thermo Fisher Scientific, Waltham, MA, USA), 100 nM dexamethasone (Merck, Darmstadt, Germany), 500 nM insulin (Merck, Darmstadt, Germany), 1 μM BRL49653 (Merck, Darmstadt, Germany), and 0.25 mM 3-isobutyl-1-methylxanthine (Merck, Darmstadt, Germany). The medium was changed every third day up to 10 days. Oil red O/Nile Red staining of neutral lipids in mature AD was performed as mentioned above.

For OB differentiation, 20 000 cells/cm^2^ were seeded and induced with OB induction medium containing 10 mM β-glycerophosphate (Merck, Darmstadt, Germany), 10 nM dexamethasone (Merck, Darmstadt, Germany), 50 μg/ml l-ascorbic acid (Wako Chemicals USA Inc., Virginia, USA), 10 nM 1,25-dihydroxyvitamin D_3_ (Merck, Darmstadt, Germany) in MEM supplemented with 10% FBS and 1% P/S. The medium was changed every third day up to 10 days. Quantification of ALP activity and Alizarin Red staining were performed as described above.

### 2.5 Micro-computed tomography (μCT) analysis

Proximal tibias and distal body of the 5th lumbar vertebra (L5) of mice fed for 8 weeks with ND, HFD or HFD supplemented with pioglitazone or MSDC-0602K were scanned with a high-resolution μCT SkyScan 1272 (Bruker, Billerica, MA, USA) with resolution 3 μm per voxel (voltage 80 kV, current 125 μA with 1 mm aluminum filter, exposure 1300 ms, 2time averaging, and 0.21° rotation step on 360°scanning). Reconstruction of virtual slices was performed in NRecon 1.7.3.1 (Bruker, Billerica, MA, USA) with InstaRecon 2.0.4.0 reconstruction engine (InstaRecon, Urbana, IL, USA) with 49% beam hardening correction, ring artifact correction = 9, and range of intensities 0–0.09 AU for tibia and 0–0.11 for L5. Reconstructions were reoriented in DataViewer 1.5.6 (Bruker, Billerica, MA, USA). Areas of interest were selected based on reference section and analyzed in CT Analyser 1.18.4.0 (Bruker, Billerica, MA, USA) with structure separation based on the Otsu's method. Cortical and trabecular bone were analyzed in the same area for structure volume, porosity, density, and connectivity. The region for the analyses was defined from the first slide under the growth plate to 230th slide. A detailed description for the quantification of 3D microarchitecture of trabecular and cortical bone has been presented previously [23].

### 2.6 Contrast-enhanced computed tomography (CECT) workflow

#### 2.6.1 Staining procedure

The current standard for studying bone marrow adipocytes (BMAds) in 3D using CECT is osmium tetroxide (OsO_4_) [24]. However, OsO_4_ is highly toxic and requires decalcification of the tissue in order to obtain reliable results and does not allow subsequent colorimetric histological staining. Recently, Kerckhofs et al. introduced a polyoxometalate (POM)-based CESA, which allows the simultaneous visualization and analysis of adipocytes, bone and blood vessels [25]. However, if a researcher is only interested in adipocytes and bone, a more efficient CESA could be used. In this study, Hexabrix, a non-toxic staining agent, was used to visualize BMAT in bones. Since bone is strongly attenuating in nature, we opted to use a CESA that was unable to interact with adipocytes, but able to generally interact with other BM constituents. This would increase the contrast differences between bone, BM and adipocytes and consequently facilitate the desired BMAT analyses. Hexabrix is well-known in the field of CECT (*in vivo* and *ex vivo*), and has extensively, but not exclusively, been used in studies concerning the visualization and quantification of sulfated glycosaminoglycans (sGAGs) in cartilage tissue. Both sGAGs and Hexabrix are negatively charged at physiological pH (7.4) and will consequently repel each other [[26], [27], [28]]. Since adipocytes contain many types of lipid molecules (free fatty acids, TG etc.) that create a hydrophobic environment containing biomolecules bearing negatively charged functional groups (phosphate and/or carboxylate groups), we expected a similar repelling effect of this CESA towards adipocytes, rendering adipocytes darker compared to the surrounding BM in the CECT images. This combined with a computed LogD (a metric for the distribution of a molecule between a hydrophobic and a hydrophilic phase at a certain pH) at pH 7.4 of −1.52 indicates that this molecule will unlikely accumulate in hydrophobic areas (e.g. BMAds). These Hexabrix-stained CECT images of BMAT were confirmed by H&E-stained sections from the same bone area and thus validating this compound to visualize BMAT.

The staining solution was prepared by mixing commercial Hexabrix® solution (Guerbet, 10 mL, 320 mgI/mL) with a 1x PBS (phosphate-buffered saline, 40 mL, 10 mM). Formalin-fixed proximal tibias (right leg) of the mice were transferred to a 1.5 mL Eppendorf tube containing 1 mL of staining solution. These Eppendorf tubes were placed on a shaker plate (gentle shaking) at ambient temperature. The samples were stained for at least 3 days, after which they were scanned.

#### 2.6.2 μCT image acquisition and reconstruction

For image acquisition, the samples were removed from the Eppendorf tube and wrapped in parafilm to prevent dehydration while exposed to X-rays. Samples were imaged using a Phoenix NanoTom M (GE Measurement and Control Solutions, Boston, MA, USA). A diamond-coated tungsten target was used. The system was operated with the following acquisition parameters: voltage = 60–70 kV, current = 120–140 μA, focal spot size = 1.99 μm, isotropic voxel size = 2 μm³, exposure time = 500 ms, frame averaging = 1, image skip = 0 and scan time = 20 min. The reconstruction was performed using Datos|x GE Measurement and Control Solutions software (version 2.7.0 – RTM) with a beam hardening correction of 8 and the inline median, ROI CT filter and Filter volume algorithms, implemented in the software. Subsequently, the datasets were normalized using an in-house developed Matlab script, with air and residual Hexabrix solution as references.

#### 2.6.3 CECT image analysis of BMAT

After consistent alignment of the datasets (DataViewer 1.5.6, Bruker MicroCT, Billerica, MA, USA), we initiated the analysis of the μCT data using CTAn (Bruker MicroCT, Billerica, MA, USA). First, we selected the volume of interest (VOI) in the proximal metaphysis of the tibia starting 250 slices below the growth plate and covering 500 slices in the distal direction. In this VOI, binarization of the dataset was performed using a global threshold (130–255). This threshold masked both cortical and trabecular bone. Based on this selection, a denoised mask for the bone marrow combined with the trabecular bone was segmented by performing a sequence of VOI shrink-wrap, closing (2–10; increments of 2) and opening (2–10; increments of 2) operations (*i.e.* everything inside the cortical bone was selected). The newly generated mask was projected on the grey value image, generating a new VOI. In this new VOI, the segmentation of adipocytes was performed using a global threshold (1–59). For the analysis of individual adipocytes, we used the Avizo 3D (version 2021.1, Thermo Fisher Scientific, Waltham, MA, USA) software. First, the adipocytes were binarized and leftover noise was removed using an interactive thresholding (1–255) and despeckle (speckle size = 7 μm × 7 μm x 7 μm) module. This was followed by the segmentation of individual adipocytes using a combination of thickness map computation and the H-extrema watershed module. Next, a border kill module was applied, which removed adipocytes that were cut by the bounding box. Then, a 3D label analysis was performed that allowed a final filtering of the data based on shape (sphericity >0.5) and volume (>4000 μm³). Finally, sphericity, volume (μm³), area (μm^2^), thickness (μm) and number of adipocytes were computed.

### 2.7 Bone strength analyses

The femora isolated from C57BL/6N male mice after 8-week-long treatment with HFD or HFD supplemented with pioglitazone and MSDC-0602K were tested in a three-point bending test using an ElectroForce testing system (TestBench LM1, EnduraTEC Systems Group, Bose Corp., Minnetonka, MN, USA). A standard protocol as described in previous work was used in this experiment [29,30]. Span length and radius of curvature of the supports were 7 mm and 2 mm, respectively. In the period between dissection and mechanical testing, the bones were fixed in 4% paraformaldehyde at 4 °C for the first 48 h after which they were stored in PBS at 4 °C. The bones were placed with the anterior surface pointing downward and were subjected to a small stabilizing preload (1 N) and two conditioning cycles before loading until failure at a rate of 0.1 mm/s. The following parameters were derived from the load–displacement curve: 1) bone strength (N), determined as the ultimate load during the three-point bending test; 2) work-to-failure (mJ), determined as the area under the load–displacement curve, representing the energy absorbed by the bone before breaking and 3) bone stiffness (N/mm), calculated as the slope of the linear proportion of the loaded-displacement curve, representing the elastic rigidity.

### 2.8 Bioenergetic analysis

Parallel measurement of oxygen consumption rate (OCR) and extracellular acidification rate (ECAR) was performed using the Seahorse XFe24 Analyzer (Agilent, Santa Clara, CA, USA). Primary mBM-MSCs were seeded in 24-well Agilent Seahorse XF Cell Culture Microplate in 5-plicates at a density of 20 000 cells per well in growth media the day prior the analysis. The next day, all wells were washed with 1 mL of DMEM (Merck, Darmstadt, Germany) supplemented with 10 mM glucose, 4 mM glutamine and 2 mM pyruvate (pH 7.4; 37 °C); 500 μL of the same media was pipetted and the microplate was incubated at 37 °C for 30 min. Meanwhile, an XFe24 sensor cartridge was prepared by injection of substrates according to the protocol [31] to measure metabolic rates with endogenous substrates (basal), and after subsequent additions with final concentration of 10 mM glucose (Merck, Darmstadt, Germany), 1 μM oligomycin (Oligo) (Merck, Darmstadt, Germany), 2 μM carbonyl cyanide-4-(trifluoromethoxy) phenylhydrazone (FCCP) (Merck, Darmstadt, Germany) and mixture of inhibitors of 1 μM rotenone (Rot) (Merck, Darmstadt, Germany), 1 μg/mL of antimycin A (AA) (Merck, Darmstadt, Germany) and 100 mM 2-deoxyglucose (2DG) (Merck, Darmstadt, Germany) (2DG + AA + Rot). During the measurement of acute effect of TZDs and MSDC-0602K on mitochondrial metabolism of human hBM-MSCs and 3T3-L1 cell line (ATCC, Washington, DC, USA) the protocol was adjusted by adding of vehicle (dimethyl sulfoxide (DMSO)), pioglitazone, rosiglitazone, MSDC-0602K or mitochondrial pyruvate carrier inhibitor - UK5099 (Merck, Darmstadt, Germany) instead of 10 mM glucose with the final concentration of 10–30 μM for TZDs and TZD analog and 2 μM for UK5099. The Seahorse data were analyzed using Wave Software 2.6.1. (Agilent, Santa Clara, CA, USA). The data were normalized by cell number determined by Hoechst 33342 staining of cell nuclei (final concentration 5 μg/mL) (Thermo Fisher Scientific, Waltham, MA, USA), which was performed immediately after the measurement using Cytation 3 Cell Imaging Reader (BioTek, Winooski, VT, USA) and processed by Gen5 software (BioTek, Winooski, VT, USA).

### 2.9 Statistical analysis

All data are representative of at least two independent experiments of similar results performed in triplicates unless otherwise indicated. The statistical significance of the differences in the means of experimental groups was determined by unpaired t-test or ANOVA and Bonferroni or Tukey post hoc tests using GraphPad Prism 5.0a software. The data are presented as means ± SEM. p value < 0.05 was considered statistically significant.

## 3 Results

### 3.1 MSDC-0602K is less detrimental than pioglitazone on bone parameters in obese mice

To determine the effect of novel TZD analog, MSDC-0602K on metabolic and bone parameters in high-fat diet (HFD)-induced obesity, C57BL/6N male mice were fed for 8 weeks with HFD or HFD supplemented with first generation of TZDs, pioglitazone (HFD + P) or MSDC-0602K (HFD + M). As shown by Bardova et al. [10] and the present study, MSDC-0602K administered to HFD mice had a similar positive impact as pioglitazone on metabolic parameters, including reduction in fasting glycemia and insulinemia and improvement of glucose tolerance (Figure. S1A–D), as well as decreased adipose tissue (AT) inflammation [10], but with a more pronounced effect with respect to reduction of weight gain (Figure. S1E–F). This may indicate less severe side effects on AT function when compared to the first generation of TZDs.

**Figure 1:**
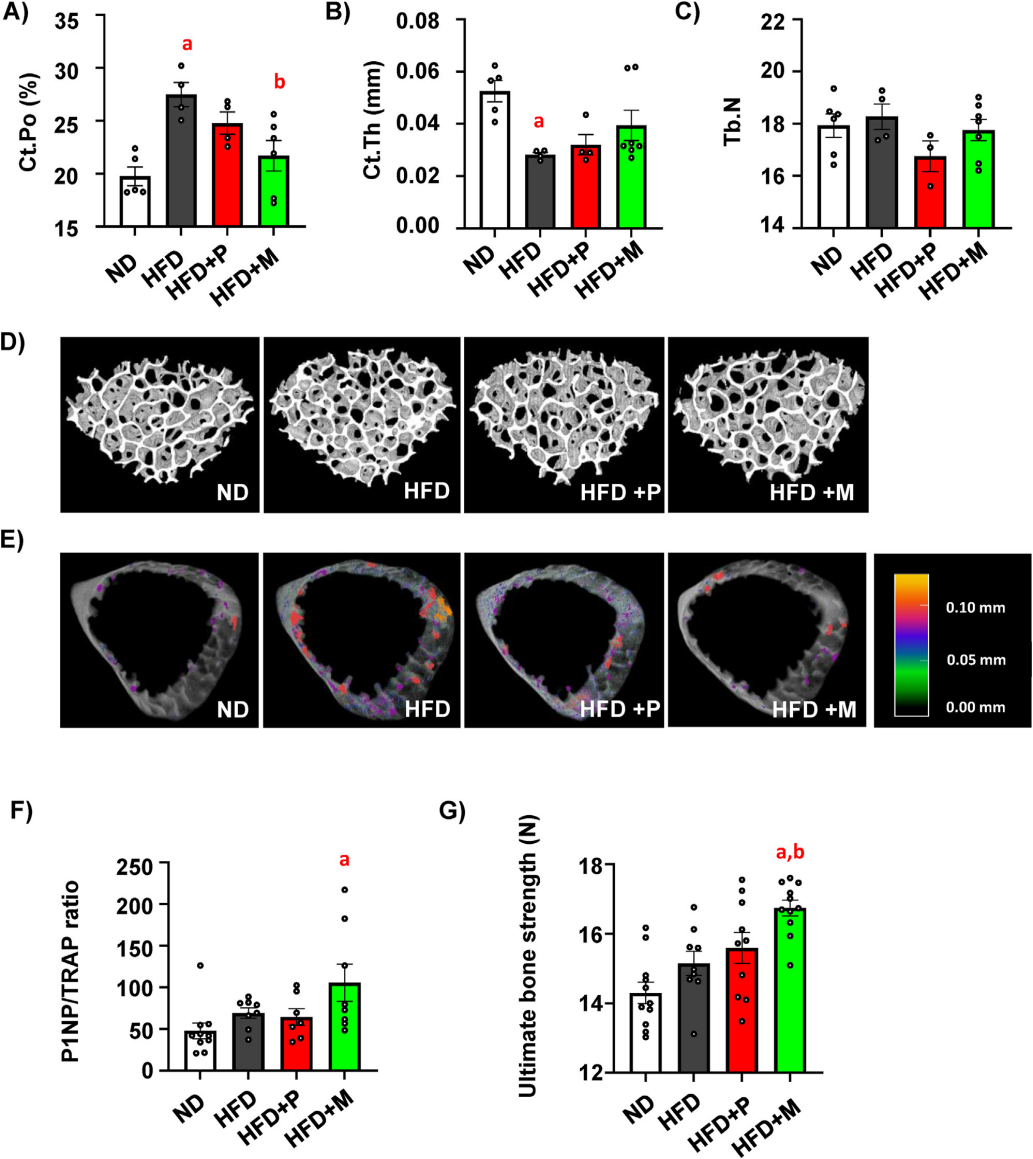
**MSDC-0602K is less detrimental than pioglitazone on bone parameters in obese mice**. (**A-E**) μCT analysis of cortical and trabecular bone in L5 vertebrae in treated mice. Cortical and trabecular parameters were calculated as (**A**) cortical porosity (Ct.Po), (**B**) cortical thickness (Ct.Th) and (**C**) trabecular number (Tb.N). Data are presented as mean ± SEM (n = 4–7 per group); one-way ANOVA, Tukey's multiple comparison test, a: ND vs other groups, b: HFD vs other groups. (**D**) Representative pictures of 3D reconstructed μCT images from trabecular and (**E**) cortical bone analysis of L5 vertebrae with colorimetric scale of pore size (scale bar 0–0.1 mm). (**F**) Analysis of the ratio of circulating markers of bone resorption (P1NP) and bone formation (TRAP) in murine plasma samples after 8 weeks of dietary intervention. Data are presented as mean ± SEM (n = 6–10 per group); one-way ANOVA, Tukey's multiple comparison test, a: ND vs other groups. (**G**) Ultimate bone strength of femurs was evaluated as first point of the plateau of the load–displacement curve measured during three-point bending test. Data are presented as mean ± SEM (n = 10–11 per group); one-way ANOVA, Tukey's multiple comparison test, a: ND vs other groups, b: HFD vs other groups. Data are presented as mean ± SEM (n = 10–11 per group); one-way ANOVA, Tukey's multiple comparison test, a: ND vs other groups.

As an extension of the above study with a focus on MSDC-0602K-induced impact on bone physiology, μCT analyses of proximal tibia and L5 vertebrae were performed in treated mice. While we did not observe significant changes in bone microstructure of proximal tibia (data not shown), there were more pronounced changes in L5 vertebrae (Figure 1A–E). HFD induced increased cortical porosity (Ct.Po) in L5, which was decreased in mice treated with HFD + M mice (Figure 1A). On the other hand, cortical thickness (Ct.Th) in L5 was decreased in HFD mice with a trend to increase in HFD + M group (Figure 1B). Further, trabecular number (Tb.N) in L5 was decreased in HFD + P compared to HFD mice, while HFD + M group showed a less detrimental effect on this parameter (Figure 1C). Other trabecular parameters were not changed (Figure. S1G–I). These changes are demonstrated in the representative 3D images of L5 trabecular and cortical bone in treated mice (Figure 1D–E). Further, ratio of circulating levels of bone formation marker procollagen type 1 N-terminal propeptide (P1NP) and bone resorption marker tartrate-resistant acid phosphatase (TRAP) (P1NP/TRAP) showed higher bone formation rate in HFD + M group compared to normal diet (ND) (Figure 1F). Bone strength measured by three-point bending test revealed stronger femora in HFD + M mice compared to HFD and HFD + P group (Figure 1G). Together these data showed that 8-week-long preventive treatment of HFD mice with MSDC-0602K reduced a negative impact on bone parameters with increased resistance to mechanical stress than typical TZD, pioglitazone.

### 3.2 BMAT composition is differently affected in obese mice treated with pioglitazone and MSDC-0602K

To further evaluate the impact of different diets on BM adiposity, the BMAT volume was analyzed using contrast-enhanced X-ray microfocus computed tomography (CECT). While μCT is unable to distinguish between distinct soft tissues due to their intrinsic low X-ray attenuation, CECT can do so by using X-ray opaque contrast-enhancing staining agents (CESAs). These CESAs enrich the X-ray attenuating atom content of the soft tissues in a specific manner. In this study, we selected Hexabrix, an anionic, iodinated CESA, which shows weaker interactions with non-adipocyte tissue constituents and is still excluded by the adipocytes. Moreover, it is a smaller molecule compared to the POM-based CESA, and thus it stains the whole tissue sample faster [[26], [27], [28]].

**Figure 2:**
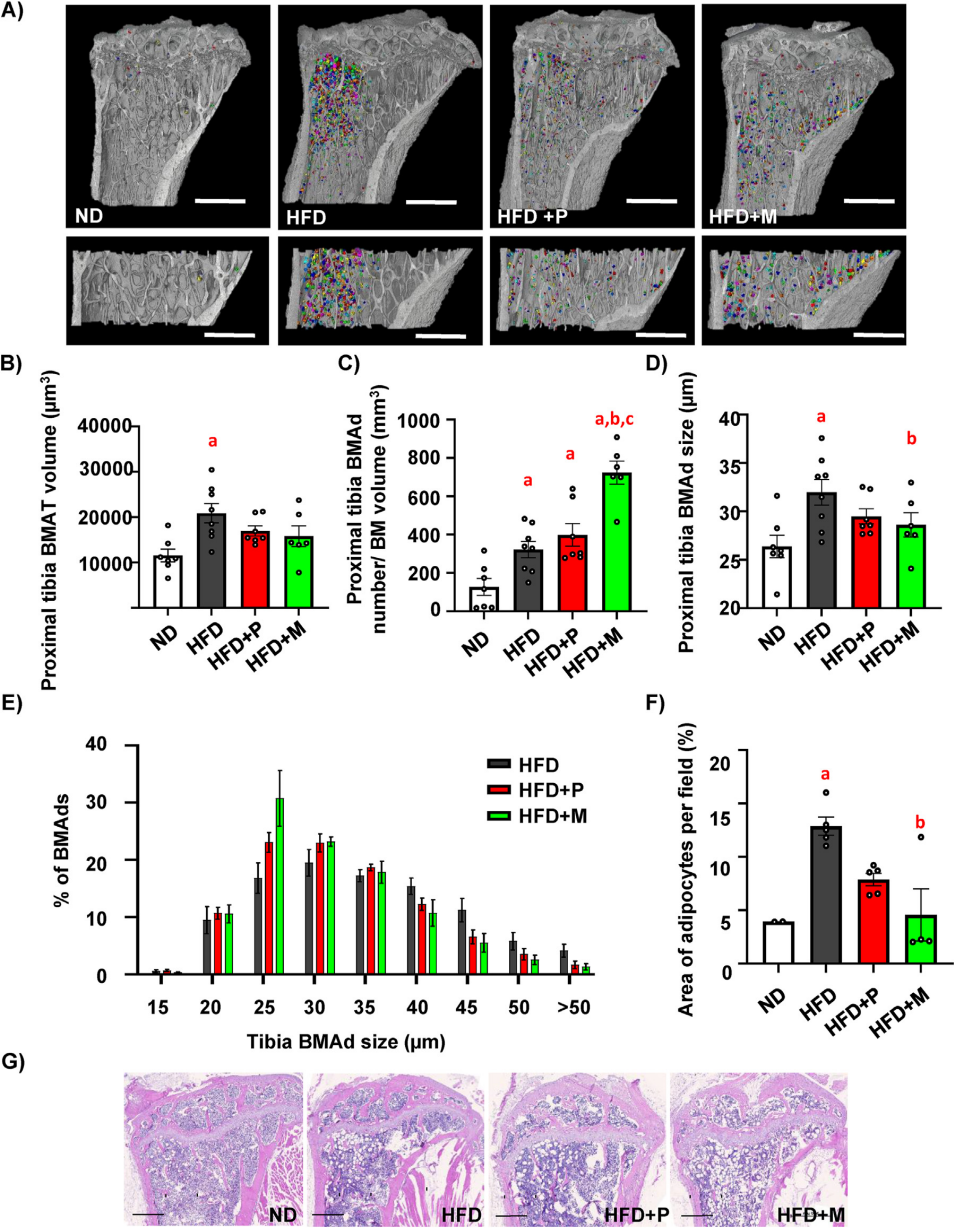
**BMAT composition is differently affected in obese mice treated with pioglitazone and MSDC-0602K**. (**A**) Representative pictures of bone marrow adipocytes (BMAds) stained with contrast agent Hexabrix in whole proximal tibia and zoomed pictures of BMAds in selected region of interest in proximal tibia (defined in Material and Methods) scanned by contrast-enhanced CT (CECT). Pictures were created using Avizo Software (scale bar 1000 μm). (**B**) Evaluation of BMAT volume in the selected region of interest in proximal tibia. (**C**) Quantification of BMAd number in the selected region of interest in proximal tibia divided by volume of selected BM region of interest (mm^3^). (**D**) Quantitative evaluation of the size of Hexabrix-stained BMAds in tibia. Data are presented as mean ± SEM (n = 6–8 per group); one-way ANOVA, Tukey's multiple comparison test, a: ND vs other groups; b: HFD vs other groups. (**E**) Analysis of different distribution of BMAd size affected by TZD and TZD analog supplementation in obese mice. (**F-G**) Histomorphometric evaluation of the BMAds expressed as (**F**) area of adipocytes per field, and (**G**) Representative pictures from H&E staining of histological section of proximal tibia from mice fed with HFD supplemented with TZD and TZD analog (scale bar 500 μm). Data are presented as mean ± SEM (n = 3–11 per group); one-way ANOVA, Tukey's multiple comparison test, a: ND vs other groups, b: HFD vs other groups.

Image analysis of Hexabrix-stained BMAds confirmed an increased BM adiposity in HFD mice as in previous studies using OsO_4_ [3,5]. A similar increase in BM adiposity was observed in the HFD + P and HFD + M groups, as depicted on the 3D representative images of BMAds in proximal tibia and BMAT volume evaluation (Figure 2A–B). The validation of the Hexabrix-stained BMAds was confirmed by the registering a cross-section of the CECT dataset with the corresponding hematoxylin-eosin (H&E)-stained section of the same tibia (Figure S2A). Interestingly, further analyses of BMAT revealed higher number of BMAds in HFD + P and HFD + M groups compared to HFD, but with a larger proportion of smaller adipocytes in HFD + M group compared to HFD + P (Figure 2C–E).

In addition, histomorphometric analysis of H&E-stained sections of proximal tibia confirmed the data from CECT, demonstrating a sustained increase in BMAds in the HFD, HFD + P and HFD + M groups compared to ND (Figure S2B), while the size of BMAds was lower in HFD + M mice (Figure 2F–G). These data highlight that MSDC-0602K treatment in HFD mice increased the proportion of smaller BMAds in tibia compared to HFD + P.

### 3.3 MSDC-0602K improves lipidome and metabolome of circulating plasma in more extend than BM composition in comparison to pioglitazone in obese mice

**Figure 3:**
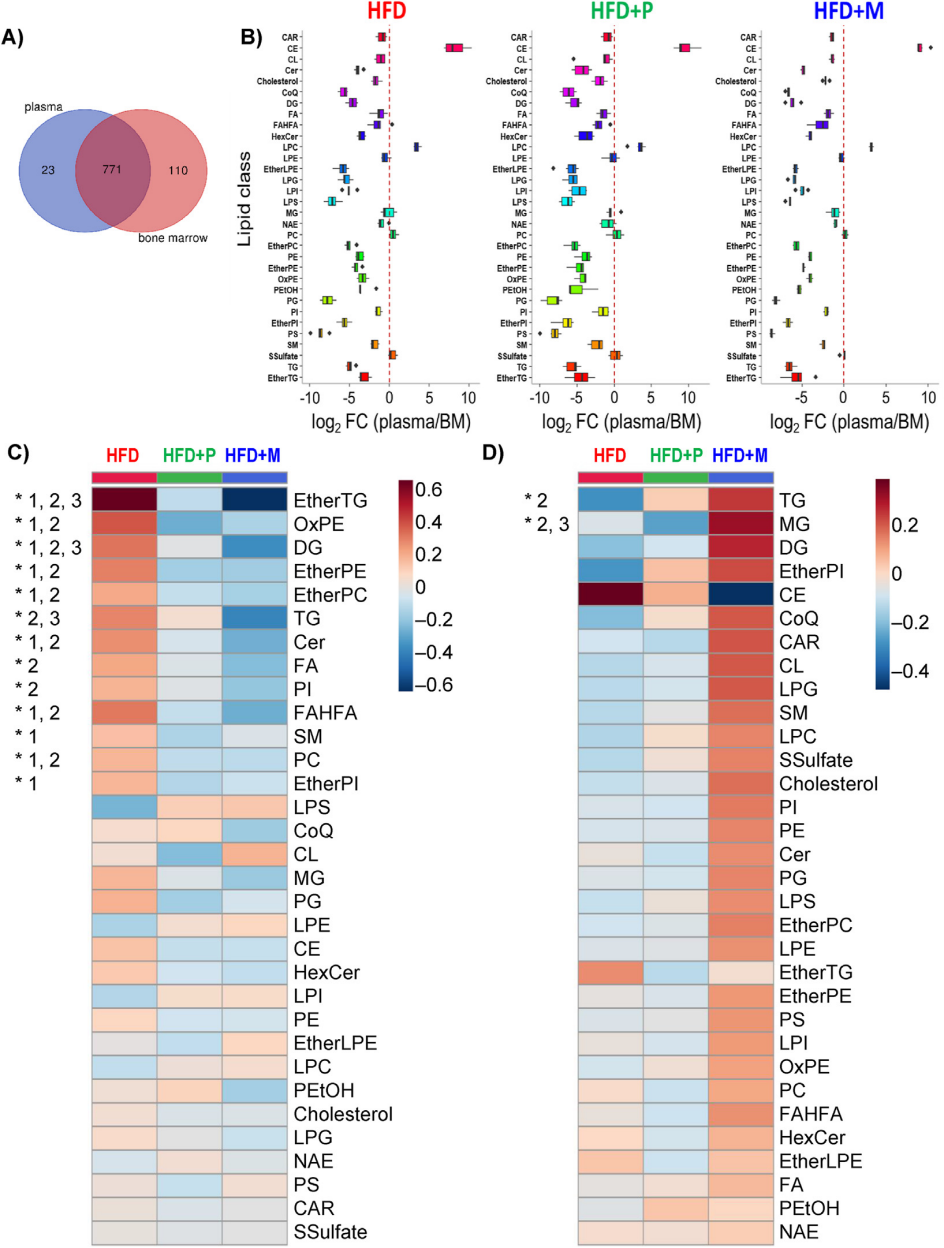
**MSDC-0602K improves lipidome and metabolome of circulating plasma in more extend than BM composition in comparison to pioglitazone in obese mice**. Global lipidomic and metabolomic analyses of plasma samples using LC–MS. **A**) Venn diagram illustrating shared and uniquely detected metabolites in plasma and BM using four LC-MS platforms. **B**) Log2-fold change of the 32 lipid classes annotated in plasma and BM in HFD, HFD + P and HFD + M groups. (**C**) Heatmap of the sum of abundances of all lipid species for each lipid class for plasma with group averages (n = 9–10 per group). Lipid classes statistically altered are marked by an asterisk (∗) based on ANOVA with p(FDR) < 0.05. Differences between groups are marked as 1 (HFD vs. HFD + P; p < 0.05), 2 (HFD vs. HFD + M; p < 0.05) and 3 (HFD + P vs. HFD + M; p < 0.05). (**D**) Heatmap of the sum of abundances of all lipid species for each lipid class for BM with group averages. Lipid classes statistically altered are marked by an asterisk (∗) based on ANOVA with p(FDR) < 0.05 (n = 6 per group). Differences between groups are marked as 2 (HFD vs. HFD + M; p < 0.05) and 3 (HFD + P vs. HFD + M; p < 0.05). (Lipid class annotation: CAR: acylcarnitine; CE: cholesteryl ester; CL: cardiolipin; Cer, ceramide; CoQ: coenzyme Q; DG: diacylglycerol; EtherPE: ether-linked phosphatidylethanolamine; EtherLPE: ether-linked lysophosphatidylethanolamine; EtherPI: ether-linked phosphatidylinositol; EtherPC: ether-linked phosphatidylcholine; EtherTG: ether-linked triacylglycerol; FA: free fatty acid; FAHFA: fatty acid ester of hydroxy fatty acid; HexCer: hexosylceramide; LPC: lysophophatidylcholine; LPE: lysophosphatidylethanolamine; LPG: lysophosphatidylglycerol; LPI: lysophosphatidylinositol; LPS: lysophosphatidylserine; MG: monoacylglycerol; NAE: N-acyl ethanolamines; OxPE: oxidized phosphatidylethanolamine; PC: phosphatidylcholine; PE: phosphatidylethanolamine; PEtOH: phosphatidylethanol; PG: phosphatidylglycerol; PI: phosphatidylinositol; PS: phosphatidylserine; SM: sphingomyelin; SSulfate: sterol sulfate; TG: triacylglycerol).

To further examine changes in systemic and BM microenvironment in treatment with different TZDs, a global lipidomic and metabolomic analysis was performed in circulating plasma and BM samples of treated mice using LC-MS. Overall, 904 polar metabolites and simple and complex lipids were annotated in these two matrices with an overlap of 85% (Figure 3A). Partial least-squares discriminant analysis (PLS-DA) showed distinct separation of HFD + P and HFD + M groups from HFD in plasma, while less separation was observed for all three groups in case of BM (Figure S3A-B). Only a few metabolites were unique or detected at a much higher intensity in plasma compared to BM (e.g., cholesteryl esters, bile acids), while BM contained more lipid classes and polar metabolites (e.g. lysophosphatidylglycerol (LPG), lysophosphatidylinositol (LPI), phosphatidylglycerol (PG), phosphatidylserine (PS), dipeptides, nucleotides), which usually maintain low concentrations in circulating plasma [32] (Figure 3B, Table S4) suggesting that these uniquely present structural components and nutrients in BM may contribute to the fate and properties of BM-MSCs. These plasma/BM ratios were not affected by the treatment with TZD drugs apart from triacylglycerol (TG) which were reduced in HFD + P and HFD + M compared to HFD group.

Next, we examined the lipidomic data from the perspective of the sum of abundances of all lipid species for each lipid class. In plasma, 13 out of 32 lipid classes were at higher concentration in HFD compared to HFD + P and HFD + M groups (Figure 3C). Specifically, the sum of ether-linked TG was detected at 3-fold and 6.5-fold higher concentrations in HFD compared to HFD + P and HFD + M, respectively, followed by other lipid classes. On the other hand, only 2 lipid classes (TG, monoacylglycerol (MG)) were statistically altered in BM. In the case of TG, their intensity was 1.5-fold higher in HFD + M compared to HFD and HFD + P (Figure 3D). Since the use of the sum of abundances may hide useful information for data interpretation, we also examined the detailed abundance patterns of all annotated lipid species along with polar metabolites. As Figure 3C shows, the top-50 metabolites in plasma belonged to lipids in line with previous data analysis with exception of lysophophatidylcholine (LPC). Among all annotated LPC species, 14 LPC species were statistically altered and formed two clusters (Figure S3E). In the first cluster, some LPC species (e.g. LPC 19:0, LPC 20:0, LPC 18:0) were increased in HFD and decreased with pioglitazone and MSDC-0602K treatment, which are known to be associated with oxidative stress and inflammation [33,34]. On the other hand, the second cluster showed the opposite trend, i.e. low concentration in HFD compared to HFD + P and HFD + M groups (e.g. LPC 15:0, LPC 14: 0), which have been shown to regulate glucose uptake in cells [34]. Contrary to plasma profile, BM showed mostly TG species among the top-50 metabolites changed with diet with trend towards higher levels in MSDC-0602K treated group (Figure S3D). Polar metabolites were not impacted with different interventions, besides three compounds including 3-hydroxybutyric acid, dimethylglycine, stachydrine associated with diet content, which were significantly altered in plasma, but they were not observed in BM. We also detected the parent drugs (pioglitazone and MSDC-0602K) present in both plasma and BM with higher concentrations in circulating plasma vs. BM.

Taken together, these data revealed that systemic treatment of obese mice with TZDs has a positive impact on decreasing circulating levels of lipids in HFD mice, while BM lipid content was changed differently, thus confirming data from μCT evaluation on different BMAT composition.

### 3.4 MSDC-0602K decreases adipocyte differentiation potential of mBM-MSCs compared to pioglitazone in obese mice

**Figure 4:**
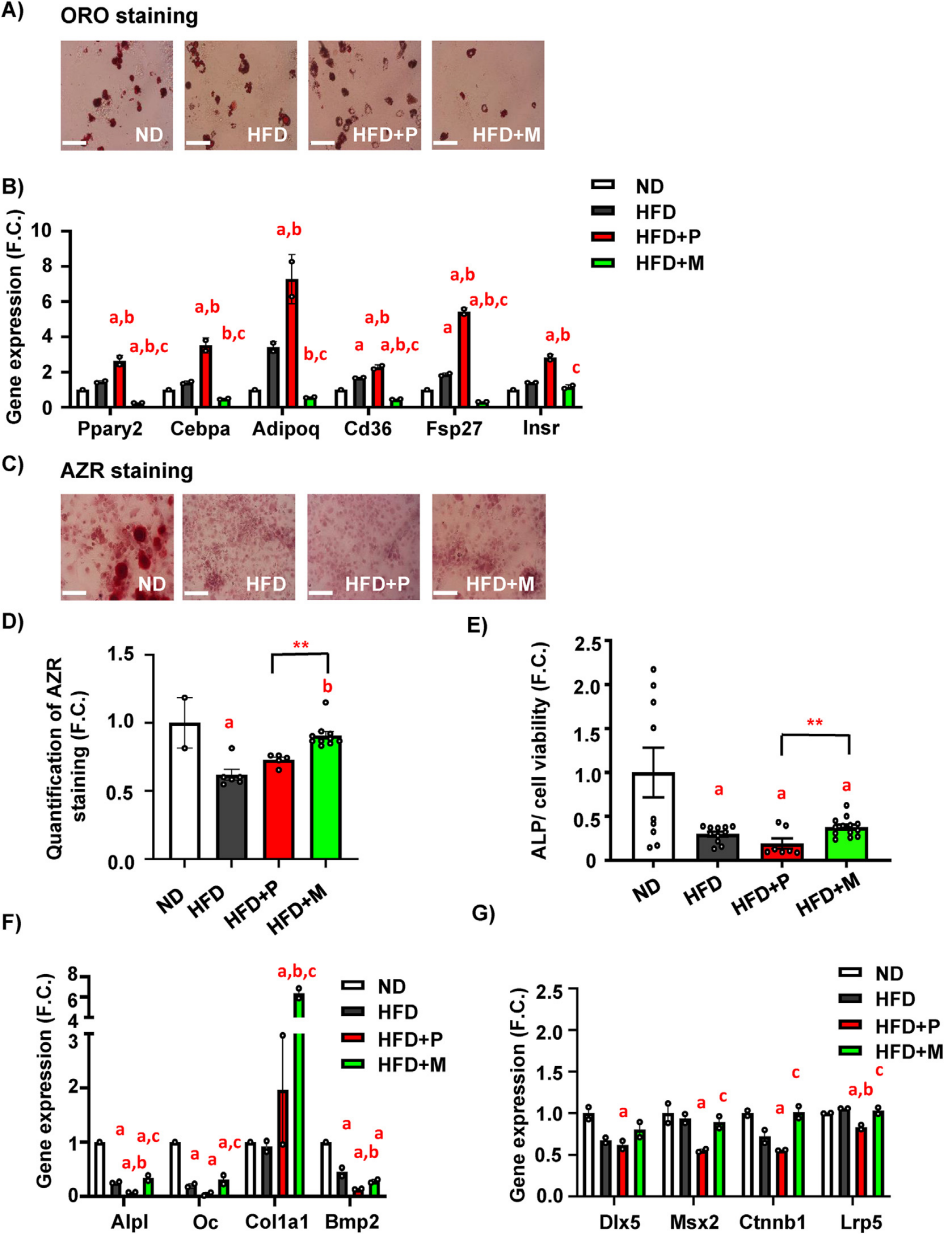
**MSDC-0602K decreases adipocyte differentiation potential of BM-MSCs compared to pioglitazone in obese mice**. (**A**) Representative picture of Oil red O (ORO) stained lipid droplets in AD differentiated mBM-MSCs (scale bar 150 μm; 20x magnification). (**B–C**) Gene expression profile of mBM-MSCs differentiated towards adipocytes in D10. (**B**) Gene expression of adipogenic genes (*Pparγ2, Cebpa, Adipoq, Cd36, Fsp27, Insr)*. Data are presented as mean fold change (F.C.) of gene expression normalized to mBM-MSCs from ND group ± SEM (n = 2 from pooled samples); one-way ANOVA, Tukey's multiple comparison test, a: ND vs other groups; b: HFD vs other groups, c: HFD + P vs other groups. (**C**) Representative pictures of Alizarin Red (AZR) staining for calcified matrix mineralization of OB differentiated mBM-MSCs (scale bar 150 μm; 20x magnification) and (**D**) quantification of eluted AZR staining of mineralized matrix in OB differentiated mBM-MSCs in D10. Data are presented as mean fold change (F.C.) of A_500_ in OB differentiated cells (OB) normalized to mBM-MSCs from ND group ± SEM (n = 2–10); one-way ANOVA, Tukey's multiple comparison test, a: ND vs other groups; b: HFD vs other groups and unpaired t-test ∗∗p < 0.01: HFD + P vs HFD + M. (n = 2–10). (**E**) Measurement of alkaline phosphatase (ALP) activity normalized to cell viability in OB differentiated mBM-MSCs in D7. Data are presented as mean fold change of ALP activity in OB differentiated cells (OB) from each experimental group ± SEM (n = 7–14); one-way ANOVA, Dunett's test comparing a: ND vs other groups and unpaired t-test ∗∗p < 0.01: HFD + P vs HFD + M. (**F**) Gene expression profile of osteoblastic markers (*Alpl, Oc, Col1a1* and *Bmp2*) and (**G**) *Dlx5, Msx2*, *Ctnnb1, Lrp5,* in OB differentiated mBM-MSCs in D10. Data are presented as mean fold change (F.C.) of gene expression normalized to mBM-MSCs from ND group ± SEM (n = 2 from pooled samples); one-way ANOVA, Tukey's multiple comparison test, a: ND vs other groups, b: HFD vs other groups, c: HFD + P vs other groups, d: HFD + M vs other groups.

To determine cellular changes related to bone formation, primary murine BM-MSCs (mBM-MSCs) were isolated from treated mice to characterize their cellular and molecular phenotype. Stem cell properties of mBM-MSCs evaluated by colony-forming units-fibroblast (CFU-f) showed increased CFU-f in HFD + P compared to HFD mice (Figure S4A–B). The short-term proliferation rate of primary cultures did not show any differences between the groups (Figure S4C). Further, as it was previously shown [3], mBM-MSCs from the HFD group manifested increased adipocyte (AD) differentiation potential compared to mBM-MSCs from the ND group, as measured by Oil Red O staining (Figure 4A). This AD differentiation potential was further elevated in HFD + P group. However, primary mBM-MSCs derived from HFD + M did not show the same pattern as their AD differentiation potential was decreased compared to HFD + P (Figure 4A). These data were confirmed by gene expression analysis of adipogenic markers (*Ppar*γ*2, Cebpa, Adipoq, Cd36, Fsp27, Insr*), which were more pronounced with pioglitazone treatment compared to MSDC-0602K treatment, suggesting higher PPARγ activity in HFD + P than HFD + M group (Figure 4B).

Osteoblast (OB) differentiation potential of primary mBM-MSCs measured by Alizarin staining (AZR) and alkaline phosphatase (ALP) activity (Figure 4C–E) revealed impairment in the HFD and HFD + P groups, while OB induction was improved in the HFD + M group. These data were also confirmed by gene expression analysis of osteoblastic genes (*Alpl, Oc, Col1a1, Bmp2, Dlx5, Msx2*) and genes involved in Wnt signaling, (*Ctnnb1, Lrp5*) (Figure 4F–G). Taken together, these data document that MSDC-0602K treatment in HFD-fed mice induces less AD potential and improves OB induction in primary mBM-MSCs compared to the first generation of TZD, pioglitazone, likely via lower activation of PPARγ.

### 3.5 MSDC-0602K increases glycolytic activity along with decreased senescence of mBM-MSCs in comparison to pioglitazone in obese mice

**Figure 5:**
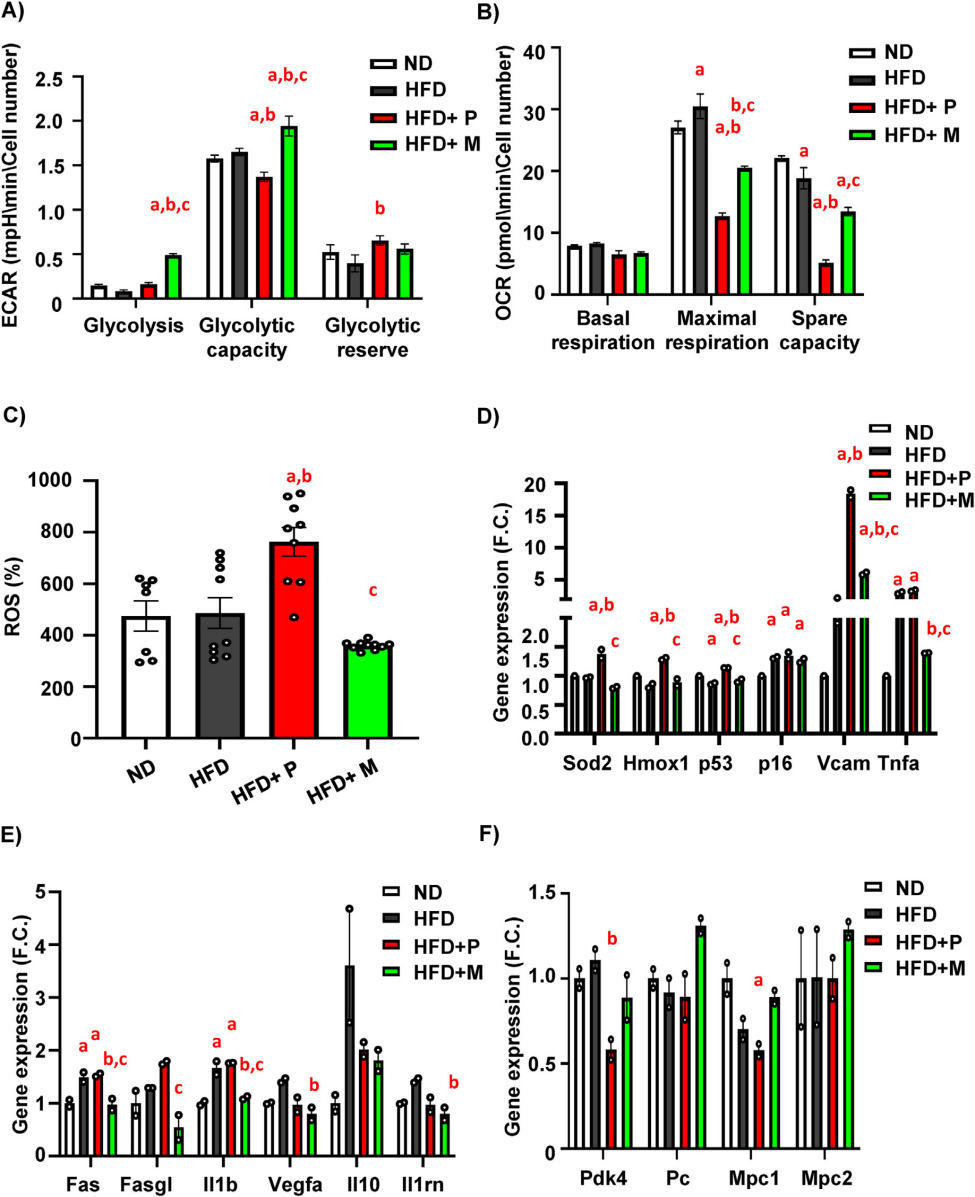
**MSDC-0602K increases glycolytic activity along with decreased senescence of mBM-MSCs in comparison to pioglitazone in obese mice**. (**A**) Glycolysis, glycolytic capacity and glycolytic reserve measured from extracellular acidification rate (ECAR) of mBM-MSCs (n = 2 independent experiments with five replicates per group). (**B**) Basal respiration, maximal respiration and spare capacity measured from oxygen consumption rate (OCR) of primary mBM-MSCs isolated from treated mice with different dietary interventions (n = 2 independent experiments with five replicates per group). Data are presented as mean ± SEM (n = 7–11 per group), one-way ANOVA, Tukey's multiple comparison test, a: ND vs other groups, b: HFD vs other groups, c: HFD + P vs other groups, d: HFD + M vs other groups. (**C**) ROS production (%) of cultivated primary mBM-MSCs isolated after 8 weeks on HFD or HFD supplemented with TZD and TZD analog. (**D**) Expression of genes associated with cell senescence (*Sod2, Hmox1, p53, p16*, *Vcam, Tnf*α) measured in differentiated mBM-MSCs (n = 2 per group from pooled samples). (**E**) Gene expression of SASP markers in primary mBM-MSCs obtained from treated mice exposed to 50uM H_2_O_2_ for 6h (*Fas, Fasgl, Il1b, Vegfa, Il10, Il1rn*) (n = 2 per group from pooled samples); (**F**) Gene expression profile of basic mitochondrial genes such as *Pdk4, Pc, Mpc1* and *Mpc2* in mBM-MSC differentiated towards osteoblasts. Data are presented as mean fold change (F.C.) of gene expression normalized to ND group ± SEM (n = 2 per group from pooled samples); one-way ANOVA, Tukey's multiple comparison test, a: ND vs other groups, b: HFD vs other groups, c: HFD + P vs other groups, d: HFD + M vs other groups.

To further understand the impact of TZD treatment on cellular metabolism, we evaluated bioenergetic profile in primary mBM-MSCs obtained from treated mice in undifferentiated state using Seahorse bioanalyzer. We obtained simultaneous measurements of mitochondrial function via the oxygen consumption rate (OCR) and glycolysis via the extracellular acidification rate (ECAR) (Figure 5).

In glycolysis measurements, primary mBM-MSCs from the HFD + M group revealed higher glycolytic capacity compared to the HFD group, while HFD + P mBM-MSCs had lower glycolytic response (Figure 5A, Figure S5A). On the other hand, measurements of cellular respiration showed higher maximal respiration rate induced by carbonyl cyanide-4-(trifluoromethoxy) phenylhydrazone (FCCP) in HFD mBM-MSCs compared to ND mBM-MSCs (Figure 5B, Figure S5B). This increase in HFD-induced OCR capacity was counteracted by treatment with pioglitazone and MSDC-0602K. Surprisingly, mBM-MSCs from HFD + M group showed less reduction of maximal OCR than those from HFD + P group (Figure 5B, Figure S5B). It might indicate that glucose is in the case of HFD + M metabolized by glycolysis and cannot fully support respiratory metabolism, which prefers other substrates.

Further, measurements of reactive oxygen species (ROS) production in primary mBM-MSCs revealed increased senescent phenotype in mice from the HFD + P group compared to HFD group (Figure 5C), while mBM-MSCs obtained from HFD + M were protected from this effect. This phenotype was also confirmed by increased expression of senescent markers (e.g. *p53*) and markers of senescence-associated secretory phenotype (SASP) (e.g. *Fas, Fasgl, Vegfa, Vcam, Tnfa, Il10, Il1rn*) [35] in primary mBM-MSCs in HFD and HFD + P groups, while mBM-MSCs in HFD + M group did not show such an increase of the selected markers (Figure 5D–E). The senescent phenotype was also manifested in primary hematopoietic stem cells (mHSCs) obtained from HFD + P group, showing increased expression of *p21, p16, Vegfa, Vcam* compared to the ND and HFD groups (Figure S5C), whereas HSCs from HFD + M mice showed less senescent features. Indeed, these findings confirmed that both mBM-MSC and mHSC populations were affected by pioglitazone and MSDC-0602K treatment, which was associated with changes in BM microenvironment. In addition, gene expression of enzymes associated with mitochondrial metabolism (*Pdk4, Pc, Mpc1 and Mpc2*) (Figure 5F) were upregulated in the HFD + M group compared to HFD + P, suggesting differential activation of mitochondria in these cells.

Taken together, these data demonstrate that MSDC-0602K differently affects cellular metabolism and nutrient utilization in mBM-MSCs compared to pioglitazone with less side effects including senescence compromising their stem cell properties.

### 3.6 MSDC-0602K reduces insulin and inflammatory responsiveness in mHSCs in comparison to pioglitazone in obese mice

As TZD treatment under diabetic conditions is known to affect immune cell function in AT by improving insulin signaling and reducing inflammatory responses [[36], [37], [38]], we investigated downstream signaling pathways related to AKT and inflammatory signaling in mHSCs (as progenitors of immune cells) isolated from HFD mice and mice fed HFD supplemented with TZDs.

**Figure 6:**
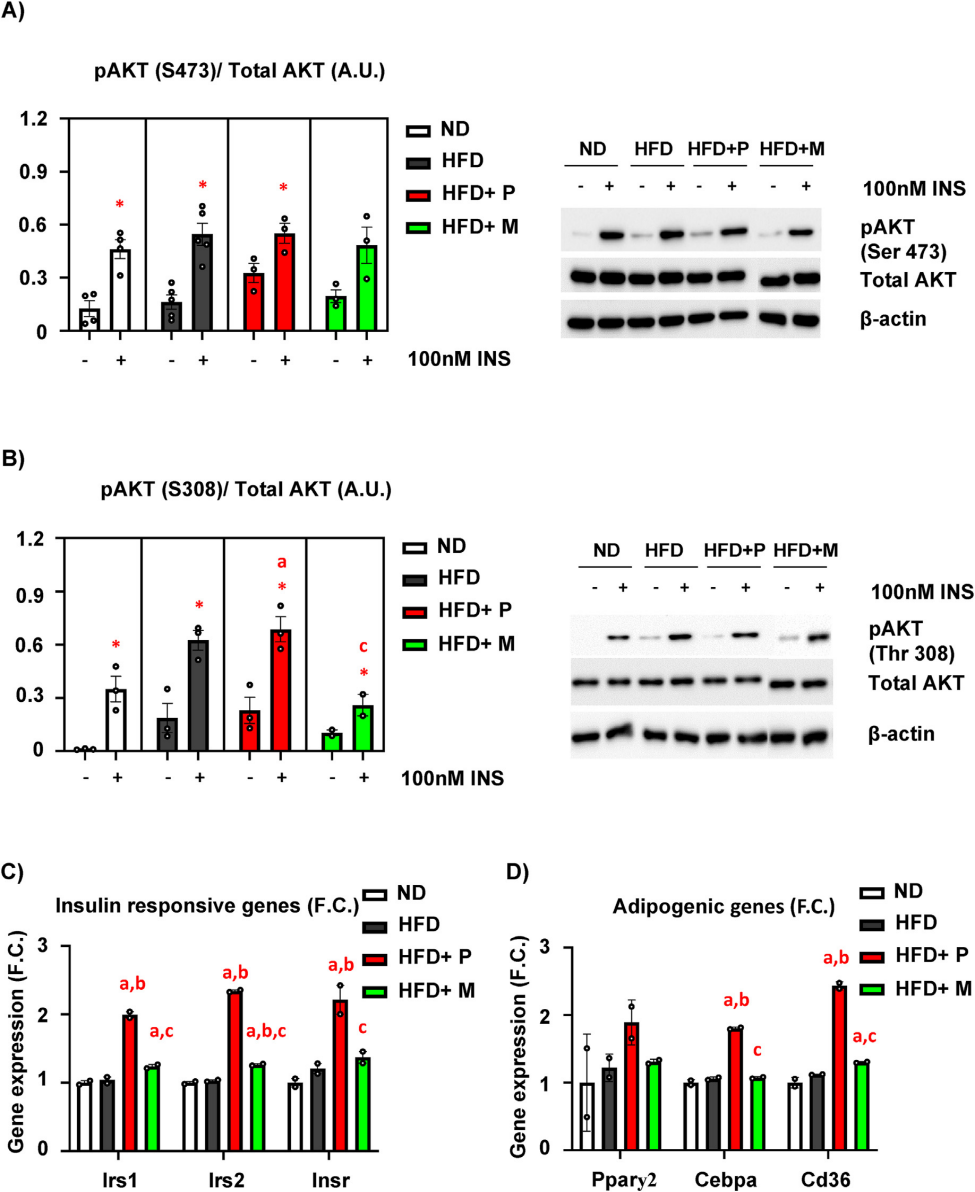
**Pioglitazone and MSDC-0602K has a different impact on mHSC insulin and inflammatory responsiveness under HFD conditions**. (**A**) Densitometry evaluation of western blot images representing the results of insulin stimulation (100 nM, 15min) of p-S473-AKT/total AKT in primary mHSCs from the ND, HFD, HFD + PIO, and HFD + M groups (n = 2–3 per group), and representative western blot images; (**B**) Densitometry evaluation of western blot images representing the results of insulin stimulation (100 nM, 15min) of p-T308-AKT/total AKT in primary mHSCs from the ND, HFD, HFD + PIO, HFD + M groups (n = 2–3 per group) and representative western blot images. Data are presented as mean densitometry ± SEM (n = 2), ∗ significant difference between –INS vs + INS (p ≤ 0.05, t-test), one-way ANOVA, Tukey's multiple comparison test, a: ND vs other groups; b: HFD vs other groups, c: HFD + P vs other groups. (**C**) Gene expression of insulin-responsive genes (*Irs1, Irs2, Insr*) and (**D**) adipogenic genes (*Pparγ2, Cebpa, Cd36*) in primary mHSCs (n = 2 per group from pooled samples) Data are presented as mean fold change (F.C.) of gene expression normalized to mHSC from ND ± SEM (n = 2 per group from pooled samples), one-way ANOVA, Tukey's multiple comparison test, a: ND vs other groups, b: HFD vs other groups, c: HFD + P vs other groups, d: HFD + M vs other groups.

Insulin stimulation measured by AKT phosphorylation (pAKT S473/total AKT, and pAKT T308/total AKT) showed that insulin signaling in primary mHSCs of HFD mice was not impaired when compared to ND cells (Figure 6A–B), similarly as in primary mBM-MSCs isolated from C57BL/6J mice [3]. In addition, pioglitazone supplementation of HFD increased insulin responsiveness in the corresponding primary mHSCs compared to the HFD and HFD + M groups mostly expressed by increased pAKT T308/total AKT (Figure 6B), which was confirmed by gene expression of insulin responsive genes (*Irs1, Irs2, Insr*) (Figure 6C). This was accompanied by increased gene expression of adipogenic genes (*Pparγ2, Cebpa, Cd36*) (Figure 6D).

In addition, gene expression of inflammatory genes (*Il1β, Tnfα, RelA*) showed increased inflammatory response in mHSCs from the HFD + P group compared to the HFD + M group (Figure S5D), suggesting that TZD has a different impact on mHSCs compared to cells in peripheral tissues. These data also correlate with other data on the PPARγ upregulation in mHSCs related to osteoclast activation and bone resorption [39], which was not present in mice receiving HFD supplemented with MSDC-0602K (Figure S5E).

### 3.7 MSDC-0602K manifests similar beneficial effects on hBM-MSC characteristics compared to classical TZDs

**Figure 7:**
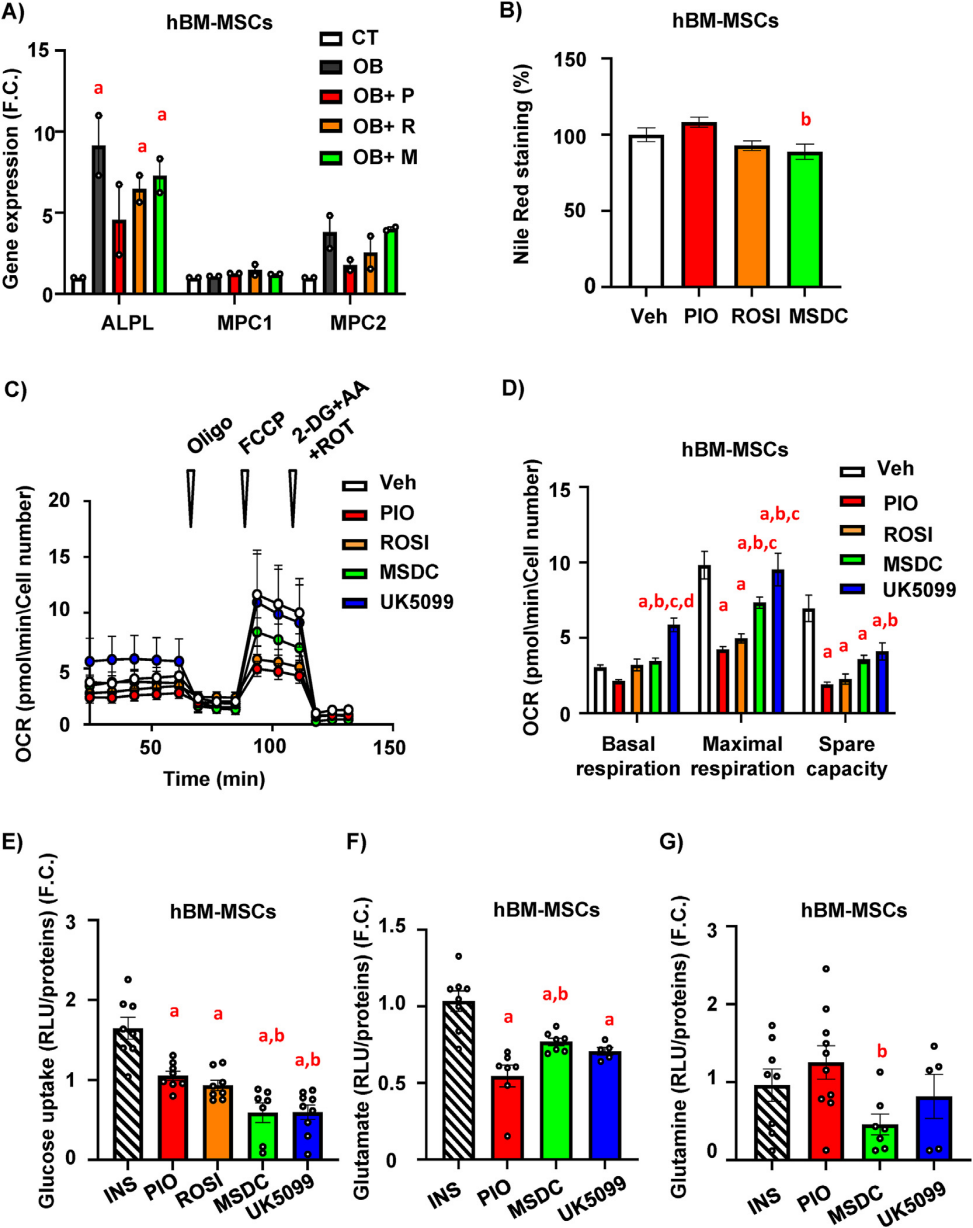
**MSDC-0602K decreases adipocyte differentiation in hBM-MSC and differently changes cellular metabolism compared to classical TZDs**. hBM-MSCs were treated for 10 days with 1 μM and 10 μM concentrations of different TZDs (rosiglitazone-ROSI, pioglitazone-PIO) or TZD analog (MSDC-0602K) together with differentiation cocktail for OB and AD differentiation. Vehicle (DMSO) was used as a control (**A**) OB differentiation of hBM-MSCs: gene expression of ALPL, MPC1, MPC2 following OB differentiation (D10) in treatment with 1uM PIO, ROSI and MSDC and vehicle (DMSO) as a control; (**B**) AD differentiation of hBM-MSCs: quantification of Nile Red staining of mature adipocytes normalized to cell viability following AD differentiation in treatment with 1 μM PIO, ROSI and MSDC and vehicle (DMSO) as a control; Data are presented as mean ± SEM from two independent experiments. One-way ANOVA, Tukey's multiple comparison test, a: Vehicle vs other groups; b: PIO vs other groups. (**C-D**) hBM-MSCs were acutely treated with 30 μM concentration of different TZDs (rosiglitazone-ROSI, pioglitazone-PIO) and TZD analog (MSDC-0602), and MPC inhibitor (UK5099) and their effect on mitochondrial respiration was analyzed. (**C-D**) Changes in oxygen consumption rate (OCR) of hBM-MSCs in response to treatment with TZDs, TZD analog and UK5099 and corresponding calculations of basal respiration, maximal respiration and spare capacity; Data are presented as mean ± SEM from at least two independent experiments with five replicates per condition; one-way ANOVA, Tukey's multiple comparison test, a: Veh vs other groups; b: PIO vs other groups, c: ROSI vs other groups, d: MSDC vs other groups. (**E**) Measurement of glucose uptake in hBM-MSC cells after 1.5-hour incubation with 1 μM INS, 30 μM PIO/ROSI/MSDC and 2 μM UK5099. Data are presented as the fold change of normalized relative light unit (RLU) value/protein of stimulated cells over non-stimulated cells. Data are presented as mean ± SEM (n = 7–11 per group); ordinary one-way ANOVA, Tukey's multiple comparison test, a: INS vs other groups; b: PIO vs other groups. Measurement of intracellular glutamate (**F**) and glutamine (**G**) in hBM-MSC cells after 3 h incubation with 1 μM INS, 30 μM PIO/MSDC and 2 μM UK5099. Data are presented as the fold change of normalized RLU value/protein of stimulated cells over non-stimulated cells. Data are presented as mean ± SEM (n = 7–11 per group); ordinary one-way ANOVA, Tukey's multiple comparison test, a: ND vs other groups.

Following *in vivo* experiment in HFD mice, we further evaluated the effect of novel TZD analog MSDC-0602K on stem cell properties in human BM-MSCs (hBM-MSCs). We used immortalized hBM-MSCs cell line described in previous studies [2,21,22,40]. OB differentiation measured by ALPL expression in hBM-MSCs showed inhibitory effect of pioglitazone and rosiglitazone treatment, which was improved by MSDC-0602K treatment (Figure 7A). Gene expression of MPC1 and MPC2 showed similar responses to different TZDs as in primary mBM-MSCs(Figure 7A). AD differentiation measured in hBM-MSCs by Nile Red staining confirmed a less stimulatory effect of MSDC-0602K when compared to first generation of TZDs (rosiglitazone and pioglitazone), similar to what was observed in mouse primary cells (Figure 7B). Further, measurement of cellular respiration in undifferentiated hBM-MSCs in response to TZDs and MSDC-0602K showed inhibitory effect of classical TZDs on maximal respiration compared to vehicle, while MSDC-0602K did not manifest such inhibition (Figure S6A–B), thus recapitulating the data from mBM-MSCs. These results confirmed the similar effects of MSDC-0602K treatment on hBM-MSCs as in mBM-MSCs, i.e. less detrimental effects on BM-MSC properties compared to typical TZDs.

### 3.8 BM-MSCs under MSDC-0602K treatment prefers glutamine over glucose utilization in comparison to AT-MSCs

Previous studies in myoblasts claimed that TZD's effect on cellular metabolism is mostly via MPC inhibition (i.e. via the inhibition of pyruvate transport) and changing substrate utilization in cells [41,42]. To further understand how TZDs and MSDC-0602K affect mitochondrial metabolism in hBM-MSCs, we analyzed how different types of TZDs affect mitochondrial function in intact cells. Our aim was to determine their inhibitory effect on the MPC pathway, the bioenergetic profile of hBM-MSCs, and the preferred energy source for mitochondrial metabolism.

Acute treatment with a lower concentration (10 μM) of TZDs and MSDC-0602K showed diminution of FCCP-induced maximal respiration compared to vehicle (Figure S7A–B), which was not present after the administration of an MPC-specific inhibitor (UK5099). This effect was even more pronounced when a higher concentration (30 μM) of tested drugs was used (Figure 7C–D). These data suggest that TZDs do not act in hBM-MSCs via inhibition of the MPC pathway as it was demonstrated in myoblasts [41].

Further, measurement of glucose uptake in hBM-MSCs after acute treatment with first generation of TZDs (pioglitazone and rosiglitazone) and MSDC-0602K revealed lower glucose uptake in cells treated with MSDC-0602K and UK5099 compared to typical TZDs (Figure 7E). On the other hand, intracellular levels of glutamate (product of glutamine metabolism after enzymatic reaction with glutaminase) were higher in MSDC-0602K-treated cells compared to pioglitazone (Figure 7F), while glutamine intracellular levels were lower (Figure 7G) suggesting preferential utilization of glutamine in cellular metabolism after administration of novel TZD analog in comparison to the first generation of TZDs.

**Figure 8:**
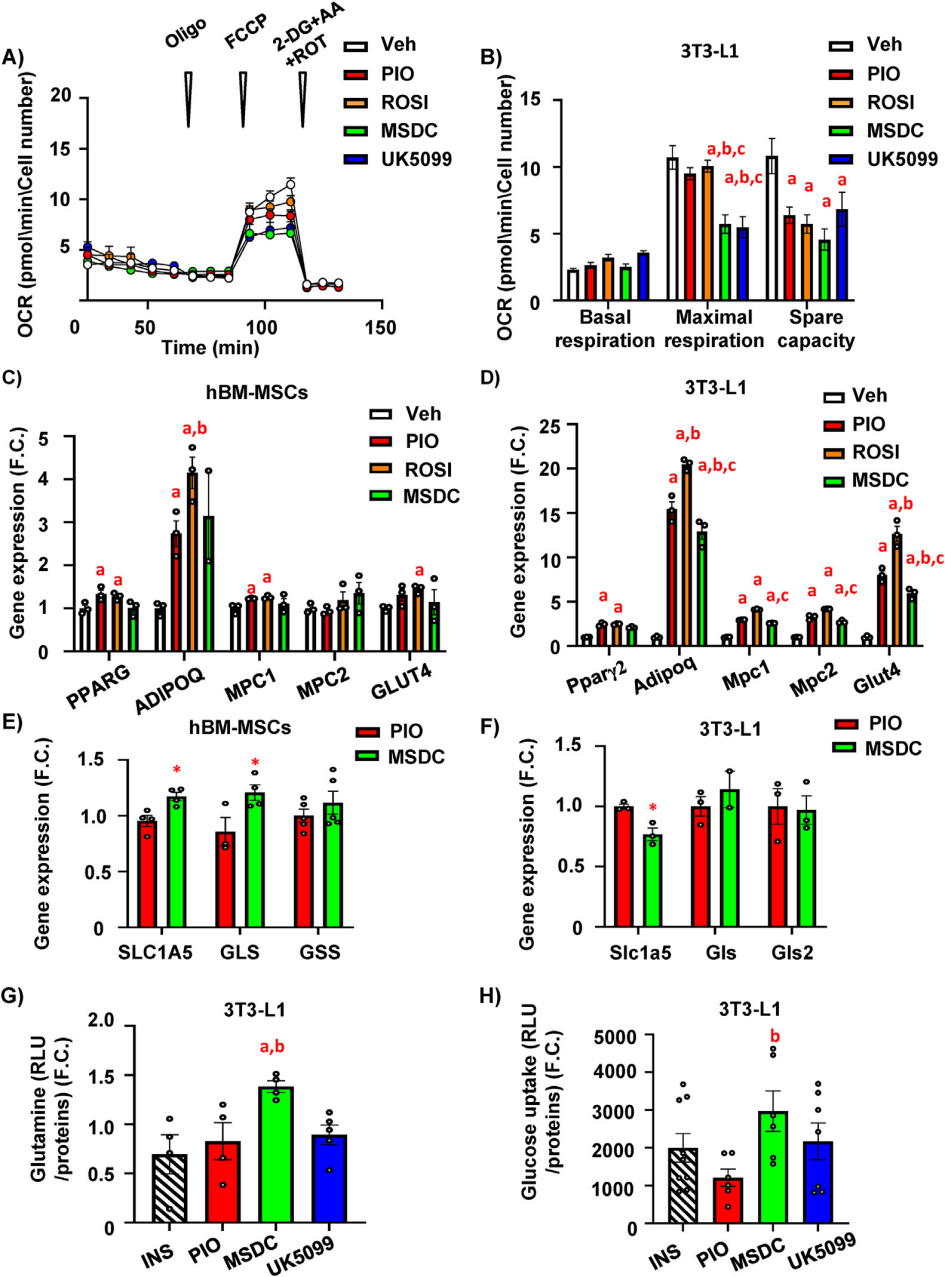
**Differential effects of TZDs and MSDC-0602K on cellular metabolism in AT-MSCs and BM-MSCs**. 3T3-L1 were acutely treated with 30 μM concentration of different TZDs (rosiglitazone-ROSI, pioglitazone-PIO) and TZD analog (MSDC-0602), and MPC inhibitor (UK5099) and their effect on mitochondrial respiration was analyzed. (**A-B**) Changes in oxygen consumption rate (OCR) of 3T3-L1 cells in response to treatment with TZDs, TZD analog and UK5099 treatment and corresponding calculations of basal respiration, maximal respiration and spare capacity. Data are presented as mean ± SEM from at least two independent experiments with five replicates per condition; one-way ANOVA, Tukey's multiple comparison test, a: Veh vs other groups; b: PIO vs other groups, c: ROSI vs other groups. (**C-D**) Gene expression profile measured in (**C**) hBM-MSC cells (*PPARG, ADIPOQ, MPC1, MPC2, GLUT4*) and (**D**) 3T3-L1 cells (*Pparγ2, Adipoq, Mpc1, Mpc2, Glut4*) differentiated to AD treated with 1 μM PIO, ROSI and MSDC-0602K. Data are presented as mean ± SEM (n = 3 per condition), one-way ANOVA Tukey's multiple comparison test, a: Veh vs other groups; b: PIO vs other groups; c: ROSI vs other groups. (**E-F**) Gene expression profiling of enzymes involved in glutamine metabolism measured in (**E**) hBM-MSCs (SLC1A5, GLS, GSS) and (**F**) 3T3-L1 cells (*Slc1a5, Gls, Gls2*). Data are presented as mean of F.C. ± SEM (n = 3–5 per condition); t-test, ∗p < 0.05: PIO vs MSDC; (**G**) Measurement of intracellular glutamine in 3T3-L1 cells after 3 h incubation with 1 μM INS, 30 μM PIO/MSDC. Data are presented as the fold change of normalized RLU value/protein of stimulated cells over non-stimulated cells. Data are presented as mean ± SEM (n = 5 per group); ordinary one-way ANOVA, Tukey's multiple comparison test, a: INS vs other groups, b: PIO vs other groups; (**H**) Measurement of glucose uptake in 3T3-L1 cells after 1.5-hour incubation with 1 μM INS, 30 μM PIO/ROSI/MSDC and 2 μM UK5099. Data are presented as the fold change of normalized RLU value/protein of stimulated cells over non-stimulated cells. Data are presented as mean ± SEM (n = 5–7 per group); ordinary one-way ANOVA, Tukey's multiple comparison test, a: INS vs other groups, b: PIO vs other groups.

To understand the effect of different TZDs on bone cells compared to peripheral tissues, we also analyzed their effect on mitochondrial metabolism in AT-MSCs (i.e. 3T3-L1). Interestingly, the lower concentration of TZDs and MSDC-0602K showed a slight increase in FCCP-induced maximal respiration compared to vehicle (Figure S7C–D), while at the higher concentration (30 μM) (Figure 8A–B), there was a significant inhibition of respiration induced by MSDC-0602K and UK5099 indicating an impact on MPC function. In addition, gene expression profiling of adipogenic and metabolic genes (*Ppar*γ*2, Cebpa, Insr, Mpc1, Mpc2*) showed differences between 3T3-L1 cells and mBM-MSCs (Figure S7E), which suggests that these cells are set up differently to respond to metabolic stimuli. AD induction assessed by gene expression profiling of adipogenic genes (*Ppar*γ*, Adipoq*) and metabolic genes (*Mpc1, Mpc2, Glut4*) showed a stronger response to TZDs in 3T3-L1 cells than in BM-MSCs (Figure 8C–D).

Next, based on the findings of the inhibitory effect of TZDs on MPC [41], we tested the hypothesis that administration of MSDC-0602K and pioglitazone may lead to differential use of nutrients to affect cell differentiation potential under *in vitro* conditions. The gene expression of markers involved in glutamine metabolism (e.g. SLC1A5, GLS) was upregulated in response to MSDC-0602K treatment of hBM-MSCs compared to pioglitazone, suggesting a shift to increased glutamine utilization by cells (Figure 8E), while no change or decrease of glutamine metabolism genes (e.g. *Slc1a5*) was observed in 3T3-L1 cells treated with MSDC-0602K compared to pioglitazone (Figure 8F). These data were further confirmed by unchanged intracellular levels of glutamate (Figure S7F) and higher levels of glutamine in MSDC-0602K-treated 3T3-L1 cells compared to pioglitazone (Figure 8G), while glucose uptake was increased in these cells (Figure 8H).

These results indicate that the TZD analog MSDC-0602K works differently on the periphery compared to BM microenvironment. These findings are important regarding further evidence of the dual role of these drugs in different organ systems.

## 4 Discussion

Treatment of obesity and type 2 diabetes with insulin-sensitizers such as TZDs has become a major challenge in clinical practice because of their side effects that include weight gain, cardiovascular complications, and increased risk of fractures [43]. In addition, many drugs prescribed for metabolic diseases have a negative effect on bone metabolism because the target molecules play dual roles in different organ systems. Here, we report that a novel TZD analog MSDC-0602K (“TZD of second generation or PPARγ-sparing TZD”), purposely designed to have a weak binding affinity to PPARγ, manifested less detrimental effects on metabolic and molecular properties of bone and mBM-MSCs in HFD-induced obesity in male mice compared with the classical TZD pioglitazone. Interestingly, we also found differential effects of MSDC-0602K on cellular metabolism in hBM-MSCs when compared to peripheral AT cells, thus confirming our hypothesis about the impact of systemically administered drugs on different tissues.

In our present study, we investigated the effects of 8-week-long administration of TZDs and a novel TZD analog MSDC-0602K on bone and energy metabolism in mice with HFD-induced obesity. The experimental design and doses of the tested drugs have been chosen according to previously published papers studying the metabolic flexibility of peripheral AT in response to dietary interventions in mice [10,18,44].

Even though previous studies in lean and HFD mice showed that of the classical TZDs, rosiglitazone has the most deleterious effect on bone loss in terms of inhibition of bone formation, increased osteoclastogenesis and induction of BMAT compared to pioglitazone [[45], [46], [47], [48], [49]], in our study we used pioglitazone in comparison to MSDC-0602K, as it is the only available PPARγ agonist used to treat T2D patients [[50], [51], [52]].

Previous studies using pioglitazone in HFD-fed male rodents (4–6 weeks) demonstrated a negative impact on vertebral bone density, decreased chondrocyte differentiation along with increased adiposity compared to non-treated animals [49,53,54]. This correlates with our data on BMAT volume, bone mechanical properties, AD differentiation potential and senescent phenotype of mBM-MSCs in HFD + P compared to HFD group. However, pioglitazone-induced detrimental effects on bone properties were not so profound compared to HFD, as in the present study we used C57BL/6N male mice and shorter dietary intervention (8 weeks vs 12–20 weeks) that did not respond to HFD administration by bone loss as is known to occur in the C57BL/6J substrain [3,5]. Nevertheless, MSDC-0602K treatment revealed a positive effect on mechanical properties in femur compared to pioglitazone treated mice, which might be explained by the changes in collagen content as OB differentiated mBM-MSCs isolated from HFD + M mice showed higher expression of *Col1a1* compared to primary cells of HFD + P mice.

Interestingly, CECT analysis of bone microstructure and BMAT content showed improved bone parameters in L5 vertebra, increased proportion of smaller BMAds in tibia along with increased mechanical properties of bones in HFD + M compared to HFD + P mice. These findings support data from Fukunaga et al. [13] demonstrating a beneficial effect of MSDC-0602K compared to rosiglitazone on bone phenotype in lean mice after 6 months of treatment.

Furthermore, our study investigated the cellular and molecular properties of primary mBM-MSCs isolated from treated mice, which has not been studied so intensively in previous reports with TZDs. Our data showed improved differentiation potential of mBM-MSCs towards OB and lower AD differentiation capacity in the HFD + M compared to HFD + P group, confirming reduced PPARγ activation in MSDC-0602K-treated mice. These changes were accompanied by less pronounced senescent and inflammatory phenotype in primary mBM-MSCs derived from mice treated with the novel TZD analog compared to the classical TZD, pioglitazone, suggesting its less detrimental effect on BM-MSC properties. Even though we did not see a decrease in lipid content measured by metabolomics in BM of HFD + M mice compared to the HFD + P and HFD group, the detailed analyses of BMAd properties by CECT revealed the impact of MSDC-0602K treatment on the distribution of smaller BMAds in the tibia, which correlated with improved bone mechanical properties. These observations may be explained by increased lipolytic activity in BMAds present in BM, which might affect the lipid cycling and using this energy to be taken by other BM-MSCs to store free fatty acids in form of small lipid droplets. This statement is further supported by decreased gene expression of metabolic genes such as *Insr*, *Ppar*γ*2, Cd36* in mBM-MSCs of HFD + M mice compared to those from HFD + P, which could correspond to a higher responsiveness to lipolytic than lipogenic signals. Based on recently published data on metabolic heterogeneity of murine and human mature adipocytes in peripheral AT using spatial and single-cell transcriptomics, one can hypothesize that MSDC-0602K treatment may affect the expansion of specific BM-MSC subpopulations with different responsiveness to insulin and thus further lead to different accumulation of lipid droplets [55,56]. Indeed, our recent study documented the presence of murine BM-MSC progenitors with a distinct metabolic program, which are differently responsive to exogenous metabolic stimuli that affect their differentiation fate [57]. However, further studies are needed to test this hypothesis using more high-throughput methods to analyze the functional heterogeneity of BM-MSCs in relation to different treatments. Regardless, our current data highlight the importance of BMAd metabolic and structural changes in response to antidiabetic drug treatment affecting bone quality.

In addition, metabolomic analysis of BM revealed the presence of unique metabolites (e.g. dipeptides, nucleotides etc.) only in BM but not in the circulation, suggesting their involvement in cellular metabolism in BM niche and affecting stem cell fate and differentiation potential. In mammals, the uptake of small peptides by the Slc15A family of oligo/dipeptide transporters provides an effective and energy-saving intracellular source of amino acids [58]. Recent study by Sharma et al. demonstrated the importance of amino acid metabolism on bone homeostasis and stem cell properties [59].

Besides MSDC-0602K, and among the novel TZDs [39,60] developed to avoid classical PPARγ transcriptional activation, Choi et al. [61] demonstrated action of a non-agonistic PPARγ ligand (SR1664) that blocks Cdk5-mediated phosphorylation at S273, thereby retaining its insulin-sensitizing effect, preventing weight gain in obese mice, and having no effect on bone formation *in vitro*. However, this compound has not been tested on bone parameters in mice with prolonged treatment. Another study by Stechschutle et al. reported that PPARγ post-translational modifications at S273 and S112 decrease PPARγ activation in resorptive osteoclasts and promote osteogenesis while maintaining its insulin-sensitizing effect [9]. Interestingly, this PPARγ agonist (SR10171) had an anabolic effect on the long bones but not on the vertebrae, whereas rosiglitazone had a negative effect in both skeletal sites explained by different cellular composition in specific skeleton sites. Our data with MSDC-0602K showed the major effect in vertebrae, which could also correlate with different cellular composition and bone turnover in vertebra vs long bones. However, we did not evaluate BMAT in vertebra site, which might have explained the different impact on bone properties.

As MSDC-0602K acts independently of PPARγ, its insulin-sensitizing effect supposes to maintain its binding affinity to mitochondria and affect cellular metabolism via MPC [11,62]. The molecular mechanism of TZD action is mainly based on the inhibition of the MPC complex (MPC1 and MPC2) [16,41]. Pyruvate uptake across the mitochondrial inner membrane is a central branch point in cellular energy metabolism with the ability to balance glycolysis and oxidative phosphorylation and control catabolic and anabolic metabolism. Transport of pyruvate into the mitochondrial matrix by the MPC is an important and rate-limiting step in its metabolism [63]. The MPC inhibition can improve cellular glucose handling, as TZDs and UK5099 increase glucose uptake in cells [41,42,[64], [65], [66]].

In our study we tested the effect of MSDC-0602K on cellular metabolism in primary mBM-MSCs, which could explain the changes in stem cell differentiation potential. Surprisingly, we did not observe an inhibitory effect of MSDC-0602K and UK5099 on mitochondrial respiration in primary mBM-MSCs isolated from treated animals, which was also confirmed in hBM-MSCs compared to pioglitazone. However, we found increased glycolytic activity in MSDC-0602K-treated cells compared to pioglitazone treatment, suggesting a change in the utilization of various nutrients contributing to the TCA cycle. This is an interesting finding as MSDC-0602K appears to act differently in peripheral AT-MSCs, in which we were able to detect an inhibitory effect of both drugs, MSDC-0602K and UK5099, on maximal respiration. Previous studies have shown that MPC activity determines the fuel oxidized by mitochondria, which has direct implications in terms of cellular functions and cell fate [67,68]. Recent findings in brown adipocytes showed that MPC inhibition leads to increased mitochondrial respiration, which activates lipid cycling and energy expenditure by replenishing energy from fatty acid and glutamine uptake [42]. However, these experiments were performed on isolated mitochondria compared to our data in intact cells.

We hypothesized that MSDC-0602K might affect glutamine metabolism as MSDC-0602K treatment increased OB induction in primary mBM-MSCs, which is associated with increased glutamine metabolism [69]. Interestingly, glucose uptake was decreased, and glutamate content (a product of glutamine metabolism) was increased in MSDC-0602K-treated BM-MSCs compared to pioglitazone. These changes were accompanied by upregulation of genes associated with glutamine metabolism in MSDC-treated cells compared to pioglitazone. Based on our findings we hypothesize that the beneficial effect of MSDC-0602K on BM-MSCs compared to AT-MSCs, is due to different preferences of nutrient utilization (i.e. glutamine over glucose uptake) in these cells, which affect their metabolic and stem cell properties supporting osteoblast differentiation in BM-MSCs but not in AT-MSCs. However, more data are needed to elucidate the exact mechanism behind the inhibitory effect of TZD on MPC and mitochondrial content and oxidative capacity, as well as its consequences in terms of changes in BM-MSC properties in relation to bone metabolism.

The present study brings several positive aspects. First, in contrast to previous studies investigating the impact of TZDs on bone, we performed a comprehensive analysis of bone and BMAd parameters along with cellular and molecular properties of primary mBM-MSCs from treated animals. Second, we applied a preventive strategy to investigate the effect of insulin-sensitizing drugs in the context of obesity and T2D. Third, we employed state-of-the-art methods to characterize cellular metabolism in BM-MSCs isolated from both mice and humans.

On the other hand, our study has some limitations. Dietary interventions in HFD-fed male mice lasted only 8 weeks, suggesting that longer treatment could have a much greater effect on bone properties. Moreover, we did not test the novel TZD analog in female mice, which is important for future perspective use of this drug in women with metabolic complications associated with osteoporosis.

Taken together, our study using HFD-induced obesity animal model shows that MSDC-0602K, as a novel insulin sensitizer, has reduced detrimental effects on bone parameters and BM-MSC phenotype by activation of glutamine metabolism as compared to the first generation of TZDs, i.e. pioglitazone. In addition, our findings provide novel insights on MSDC-0602K action *in vivo* and thus might further contribute to more specialized strategies in treatment of both bone and metabolic diseases.

## Author contributions

MT and JK conceived the project. AB, MF, MT, KB, JF, GA, MD, MR and JK designed *in vivo* experiments, performed the *in vivo and in vitro* experiments, collected and analyzed data. AP and TM provided material and helped with interpretation of bioenergetic profiling results. TC performed and helped with interpretation of metabolomics data. TB and GK performed BMAT measurement in bones *ex vivo* and helped with BMAT evaluation, and data interpretation. WW and GHL helped performed three-point bending tests and data interpretation. JP and FS performed μCT scanning of bones *ex vivo* and helped with the data analysis. MT, AB, MR and JK designed and supervised the study and wrote the manuscript. All authors revised and approved the manuscript.

## Data Availability

Data will be made available on request.
