# Supplementary material for: Novel thiazolidinedione analog reduces a negative impact on bone and mesenchymal stem cell properties in obese mice compared to classical thiazolidinediones
Source: Mol Metab. 2022 Sep 11;65:101598. doi: 10.1016/j.molmet.2022.101598 (PMC9508355; doi:10.1016/j.molmet.2022.101598)
Supplement: Multimedia component 1 [file mmc1.ppt]

## Slide 1
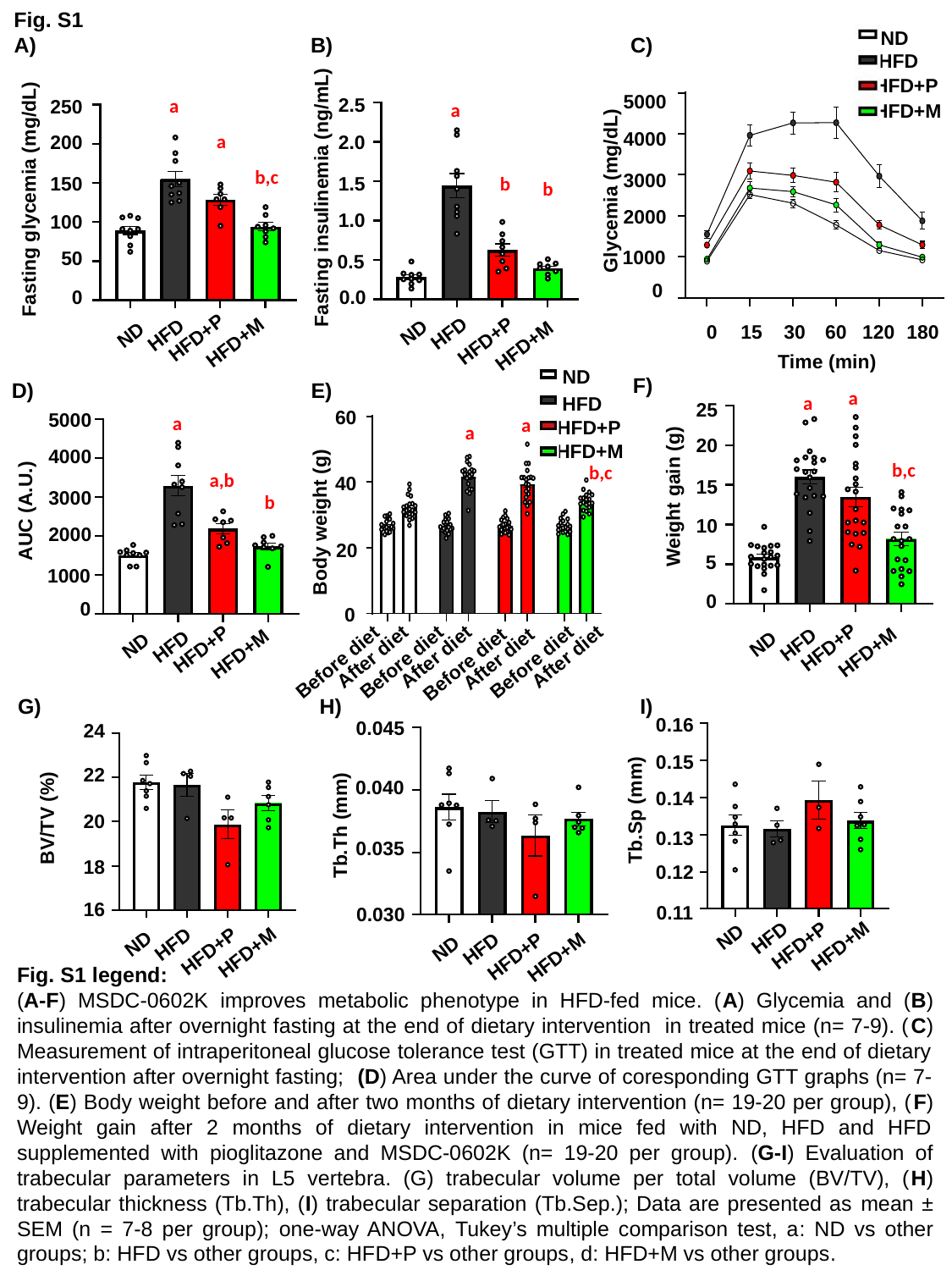

Fig. S1
ND
HFD
HFD+P
5000
HFD+M
4000
3000
Glycemia (mg/dL)
2000
1000
0
0
15
30
60
120
180
A)
B)
C)
2.5
a
2.0
b
1.5
b
Fasting insulinemia (ng/mL)
1.0
0.5
0.0
ND
HFD
HFD+P
HFD+M
a
250
a
200
b,c
150
Fasting glycemia (mg/dL)
100
50
0
ND
HFD
HFD+P
HFD+M
Time (min)
ND
HFD
60
HFD+P
HFD+M
40
20
0
After diet
After diet
After diet
After diet
Before diet
Before diet
Before diet
Before diet
Body weight (g)
a
a
b,c
F)
D)
E)
a
a
25
20
b,c
15
Weight gain (g)
10
5
0
ND
HFD
HFD+P
HFD+M
5000
a
4000
a,b
3000
b
AUC (A.U.)
2000
1000
0
ND
HFD
HFD+P
HFD+M
G)
H)
I)
0.16
0.15
0.14
Tb.Sp (mm)
0.13
0.12
0.11
ND
HFD
HFD+P
HFD+M
0.045
0.040
Tb.Th (mm)
0.035
0.030
ND
HFD
HFD+P
HFD+M
24
22
BV/TV (%)
20
18
16
ND
HFD
HFD+P
HFD+M
Fig. S1 legend:
(A-F) MSDC-0602K improves metabolic phenotype in HFD-fed mice. (A) Glycemia and (B) insulinemia after overnight fasting at the end of dietary intervention in treated mice (n= 7-9). (C) Measurement of intraperitoneal glucose tolerance test (GTT) in treated mice at the end of dietary intervention after overnight fasting; (D) Area under the curve of coresponding GTT graphs (n= 7-9). (E) Body weight before and after two months of dietary intervention (n= 19-20 per group), (F) Weight gain after 2 months of dietary intervention in mice fed with ND, HFD and HFD supplemented with pioglitazone and MSDC-0602K (n= 19-20 per group). (G-I) Evaluation of trabecular parameters in L5 vertebra. (G) trabecular volume per total volume (BV/TV), (H) trabecular thickness (Tb.Th), (I) trabecular separation (Tb.Sep.); Data are presented as mean ± SEM (n = 7-8 per group); one-way ANOVA, Tukey’s multiple comparison test, a: ND vs other groups; b: HFD vs other groups, c: HFD+P vs other groups, d: HFD+M vs other groups.

## Slide 2
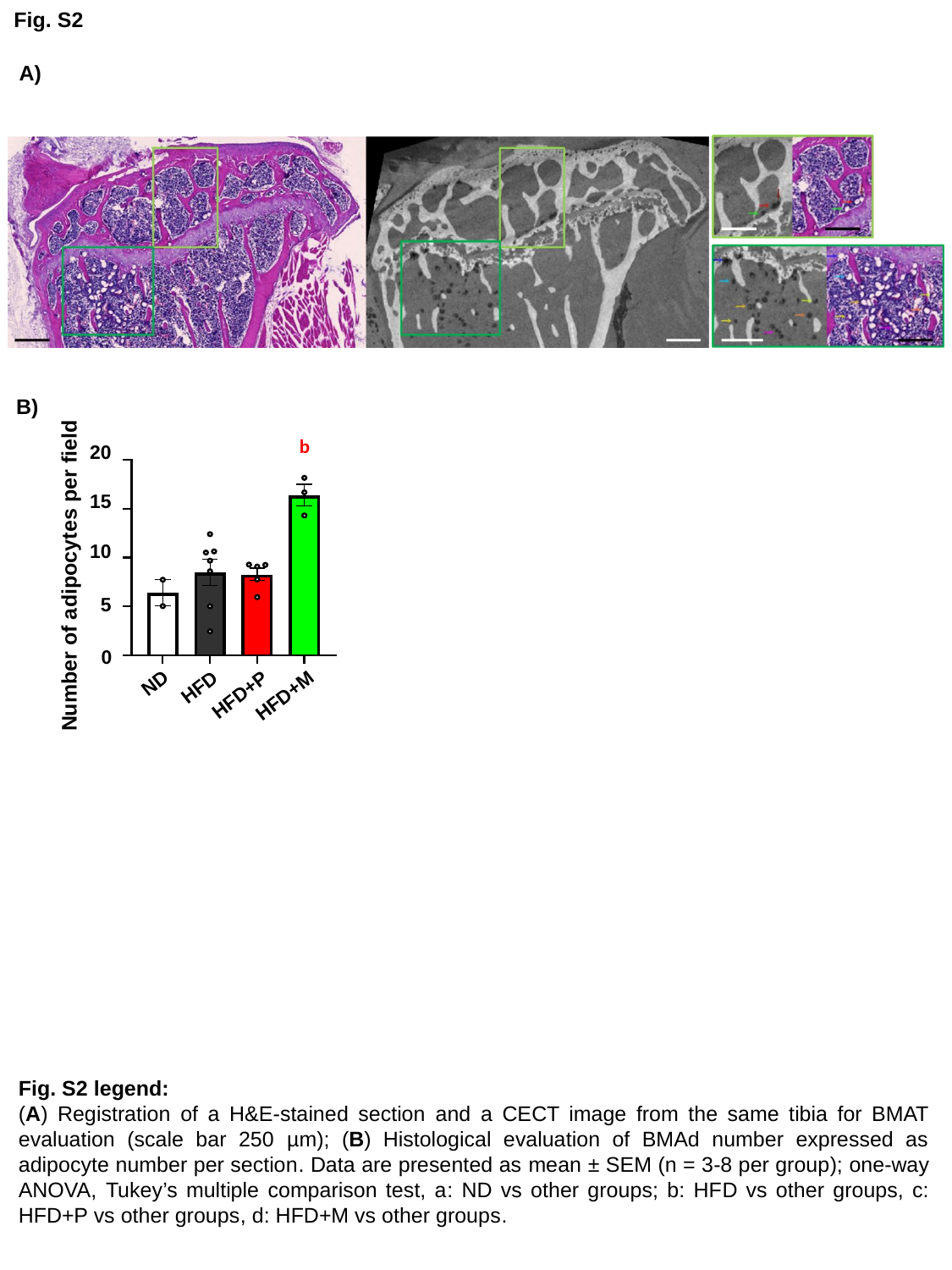

Fig. S2
A)
b
20
15
10
Number of adipocytes per field
5
0
ND
HFD
HFD+P
HFD+M
B)
Fig. S2 legend:
(A) Registration of a H&E-stained section and a CECT image from the same tibia for BMAT evaluation (scale bar 250 µm); (B) Histological evaluation of BMAd number expressed as adipocyte number per section. Data are presented as mean ± SEM (n = 3-8 per group); one-way ANOVA, Tukey’s multiple comparison test, a: ND vs other groups; b: HFD vs other groups, c: HFD+P vs other groups, d: HFD+M vs other groups.

## Slide 3
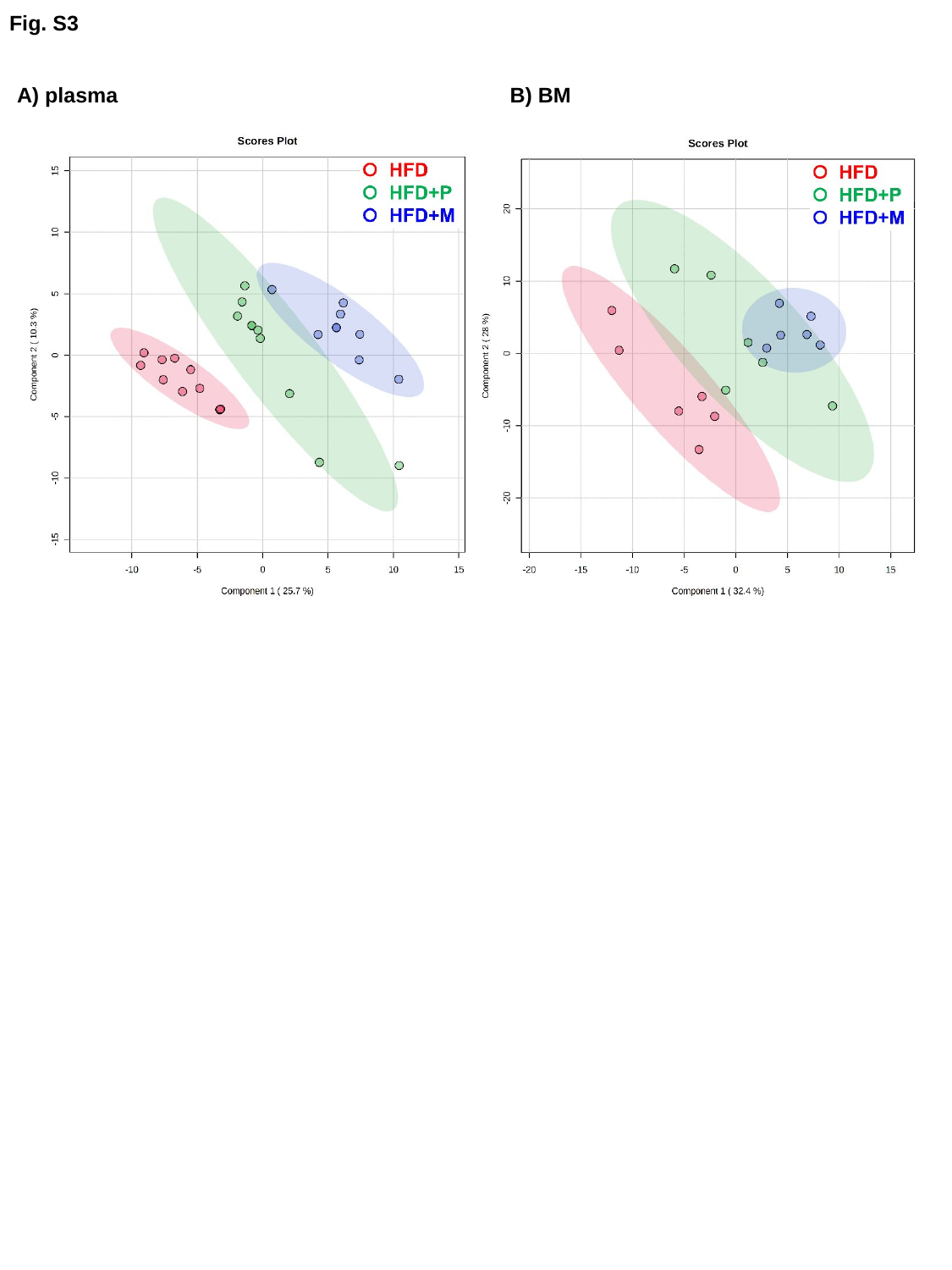

Fig. S3
A) plasma
B) BM

## Slide 4
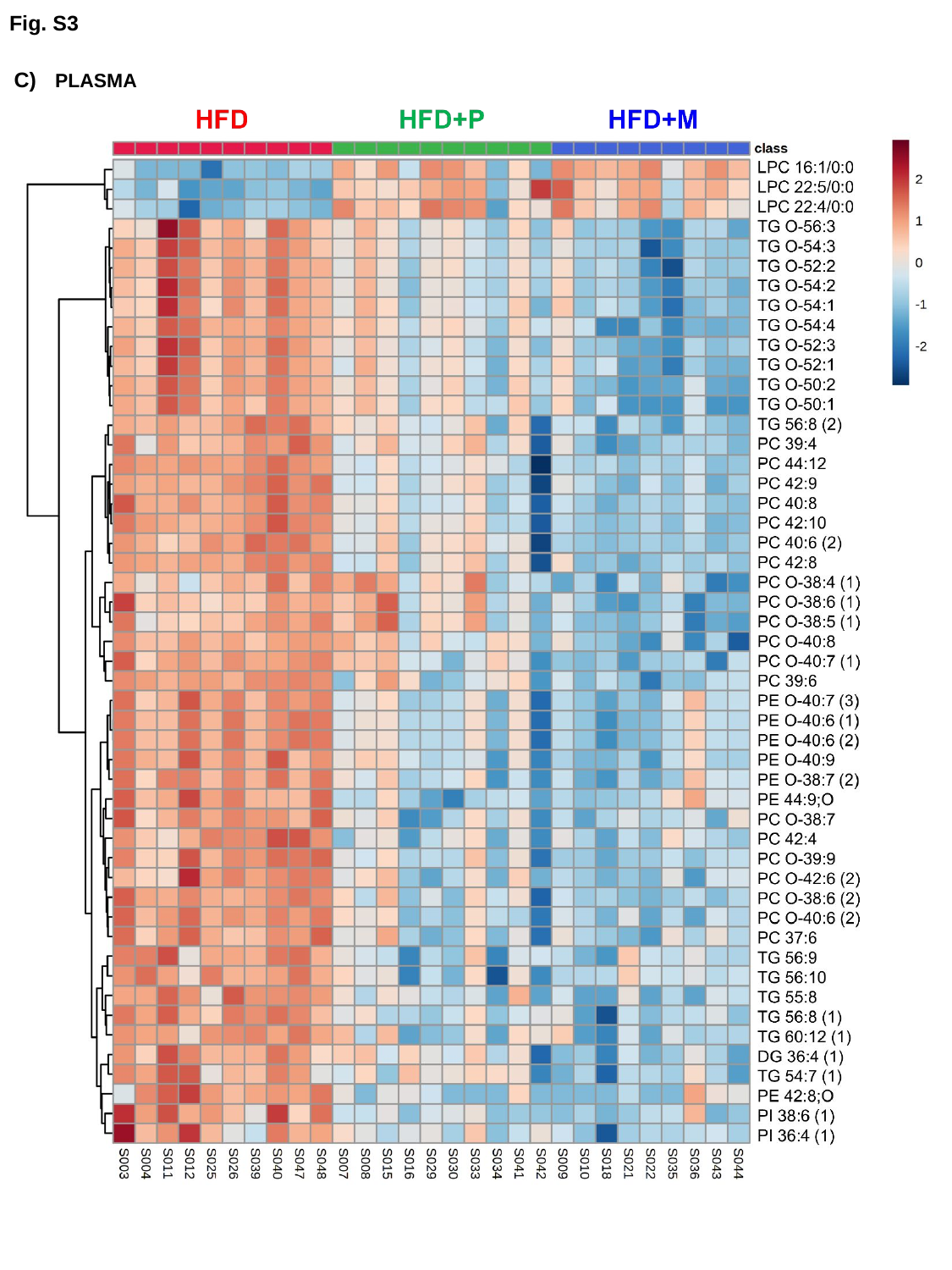

Fig. S3
C)
PLASMA

## Slide 5
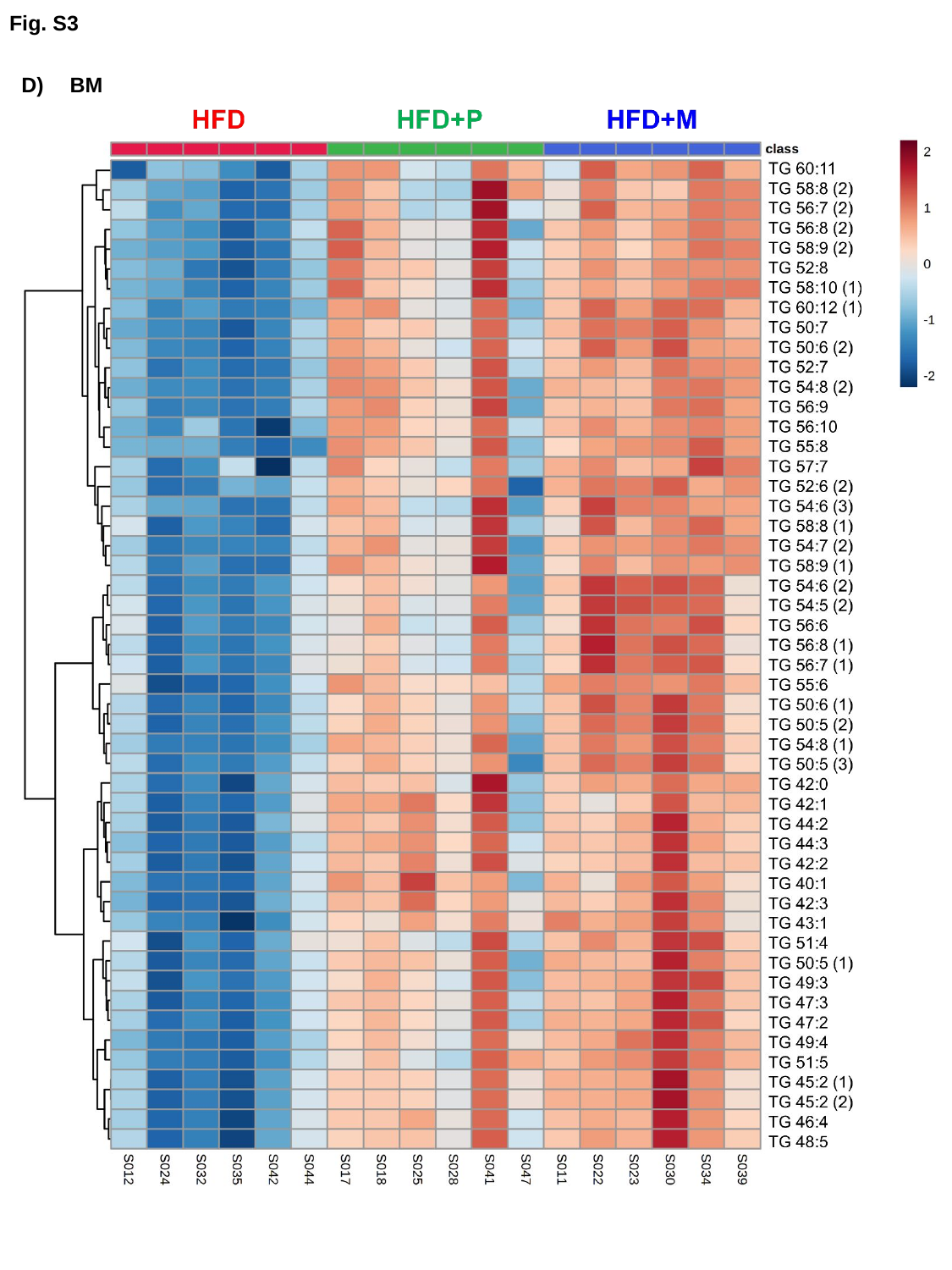

Fig. S3
D)
BM

## Slide 6
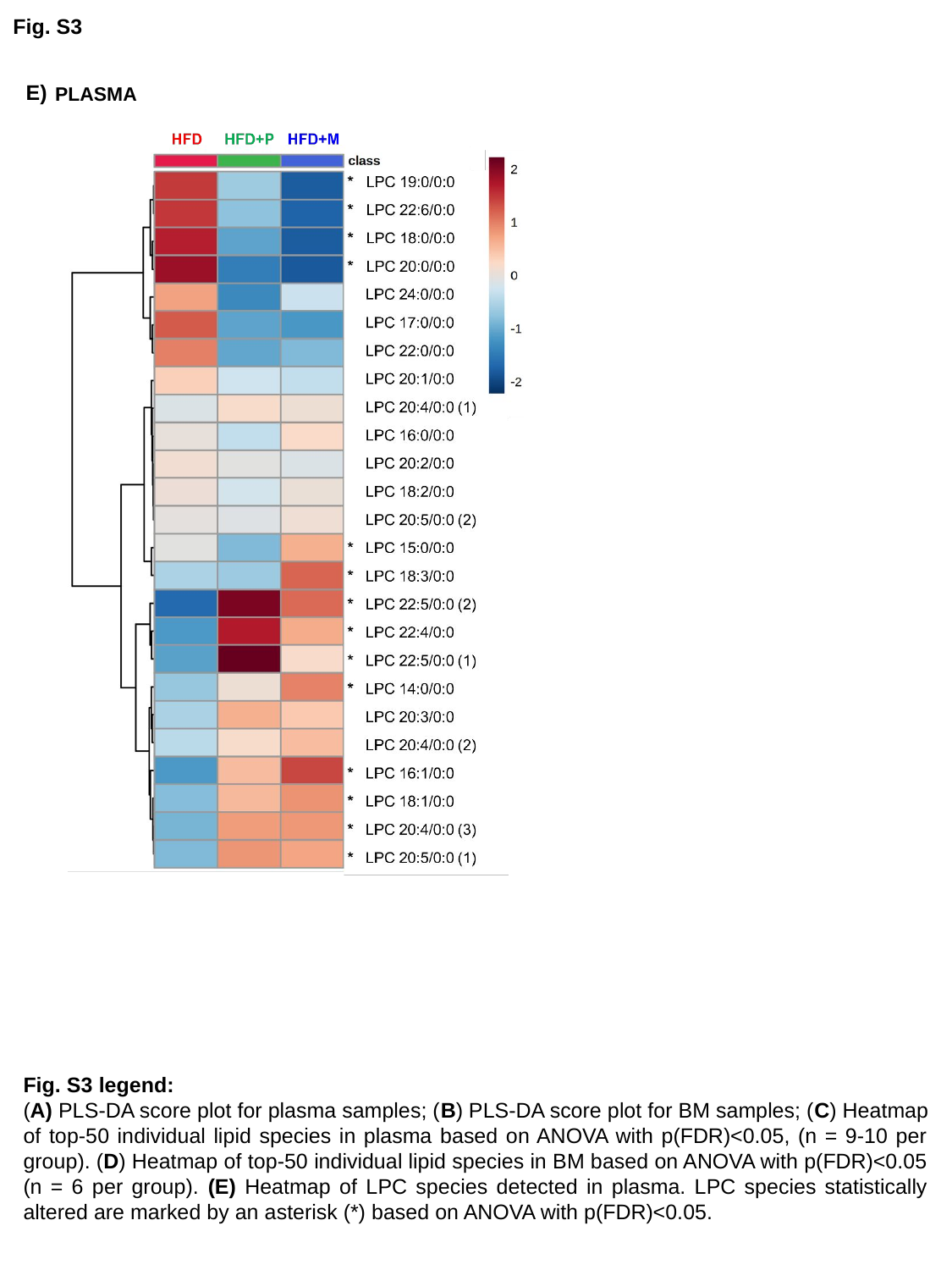

Fig. S3
E)
PLASMA
ND
HFD
Fig. S3 legend:
(A) PLS-DA score plot for plasma samples; (B) PLS-DA score plot for BM samples; (C) Heatmap of top-50 individual lipid species in plasma based on ANOVA with p(FDR)<0.05, (n = 9-10 per group). (D) Heatmap of top-50 individual lipid species in BM based on ANOVA with p(FDR)<0.05 (n = 6 per group). (E) Heatmap of LPC species detected in plasma. LPC species statistically altered are marked by an asterisk (*) based on ANOVA with p(FDR)<0.05.

## Slide 7
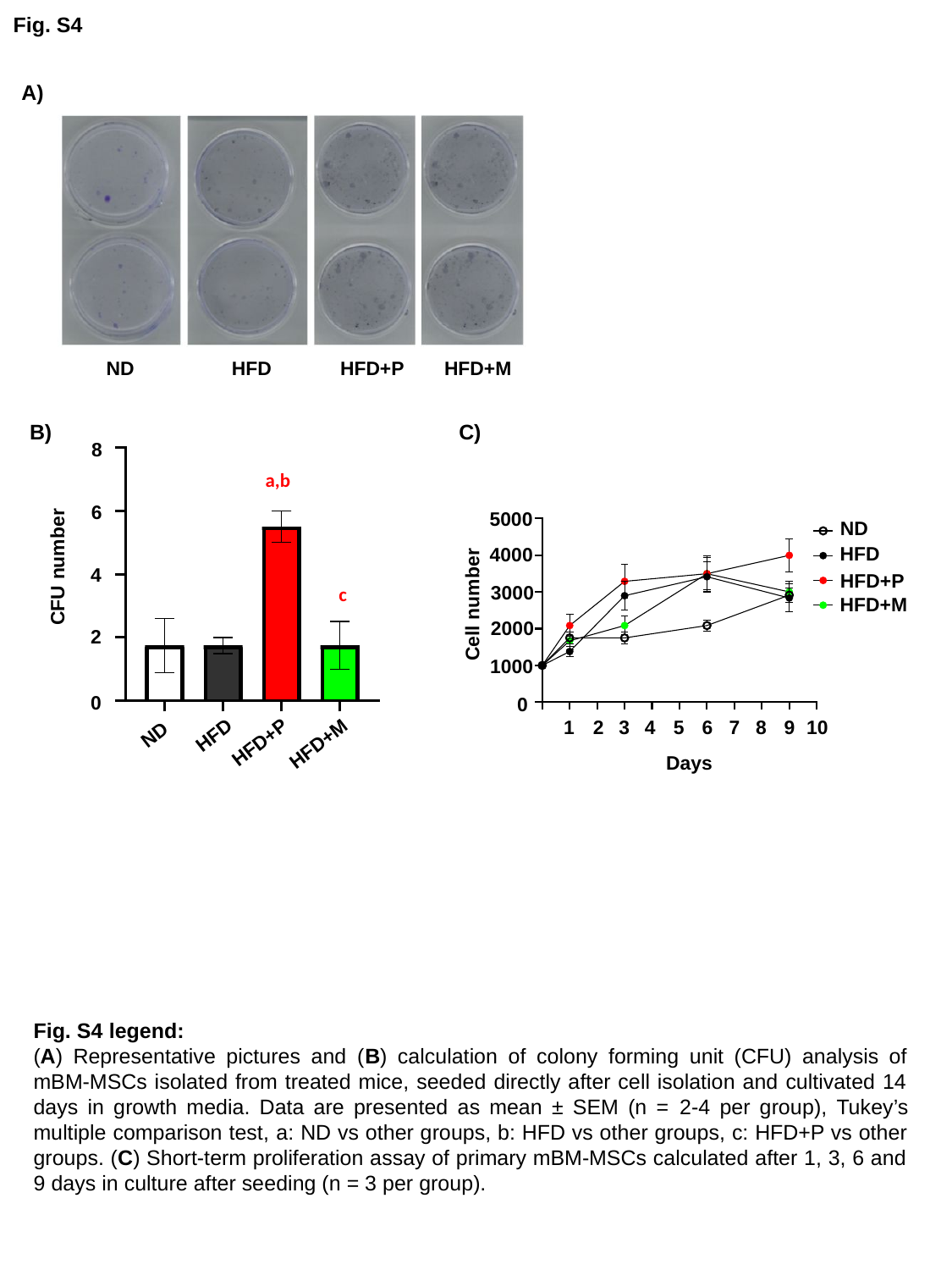

Fig. S4
A)
ND
HFD+P
HFD+M
HFD
8
a,b
6
CFU number
4
c
2
0
ND
HFD
HFD+P
HFD+M
B)
C)
5000
ND
HFD
4000
HFD+P
3000
HFD+M
Cell number
2000
1000
0
1
2
3
4
5
6
7
8
9
10
HFD
Days
Fig. S4 legend:
(A) Representative pictures and (B) calculation of colony forming unit (CFU) analysis of mBM-MSCs isolated from treated mice, seeded directly after cell isolation and cultivated 14 days in growth media. Data are presented as mean ± SEM (n = 2-4 per group), Tukey’s multiple comparison test, a: ND vs other groups, b: HFD vs other groups, c: HFD+P vs other groups. (C) Short-term proliferation assay of primary mBM-MSCs calculated after 1, 3, 6 and 9 days in culture after seeding (n = 3 per group).

## Slide 8
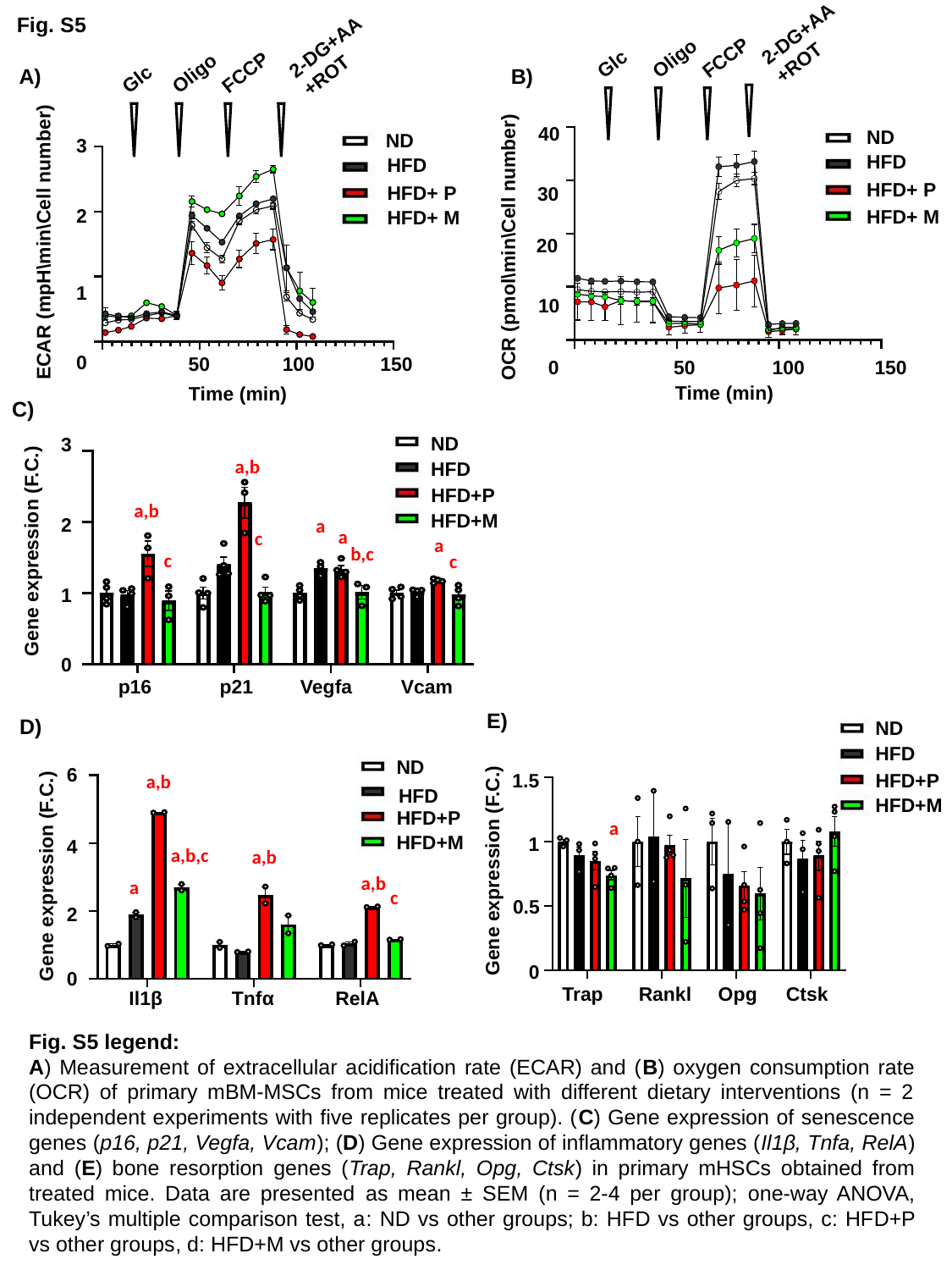

Fig. S5
2-DG+AA
+ROT
2-DG+AA
+ROT
Glc
Oligo
FCCP
Oligo
Glc
FCCP
A)
B)
40
ND
ND
3
HFD
HFD
HFD+ P
HFD+ P
30
2
HFD+ M
HFD+ M
ECAR (mpH\min\Cell number)
OCR (pmol\min\Cell number)
20
1
10
0
50
100
150
0
50
100
150
Time (min)
Time (min)
C)
ND
3
2
1
0
a,b
HFD
HFD+P
a,b
HFD+M
a
a
c
a
Gene expression (F.C.)
b,c
c
c
p16
p21
Vegfa
Vcam
E)
D)
ND
HFD
ND
6
HFD
HFD+P
HFD+M
4
Gene expression (F.C.)
2
0
Il1β
Tnfα
RelA
a,b
a,b,c
a,b
a,b
a
1.5
HFD+P
HFD+M
a
1
Gene expression (F.C.)
c
0.5
0
Trap
Rankl
Opg
Ctsk
Fig. S5 legend:
A) Measurement of extracellular acidification rate (ECAR) and (B) oxygen consumption rate (OCR) of primary mBM-MSCs from mice treated with different dietary interventions (n = 2 independent experiments with five replicates per group). (C) Gene expression of senescence genes (p16, p21, Vegfa, Vcam); (D) Gene expression of inflammatory genes (Il1β, Tnfa, RelA) and (E) bone resorption genes (Trap, Rankl, Opg, Ctsk) in primary mHSCs obtained from treated mice. Data are presented as mean ± SEM (n = 2-4 per group); one-way ANOVA, Tukey’s multiple comparison test, a: ND vs other groups; b: HFD vs other groups, c: HFD+P vs other groups, d: HFD+M vs other groups.

## Slide 9
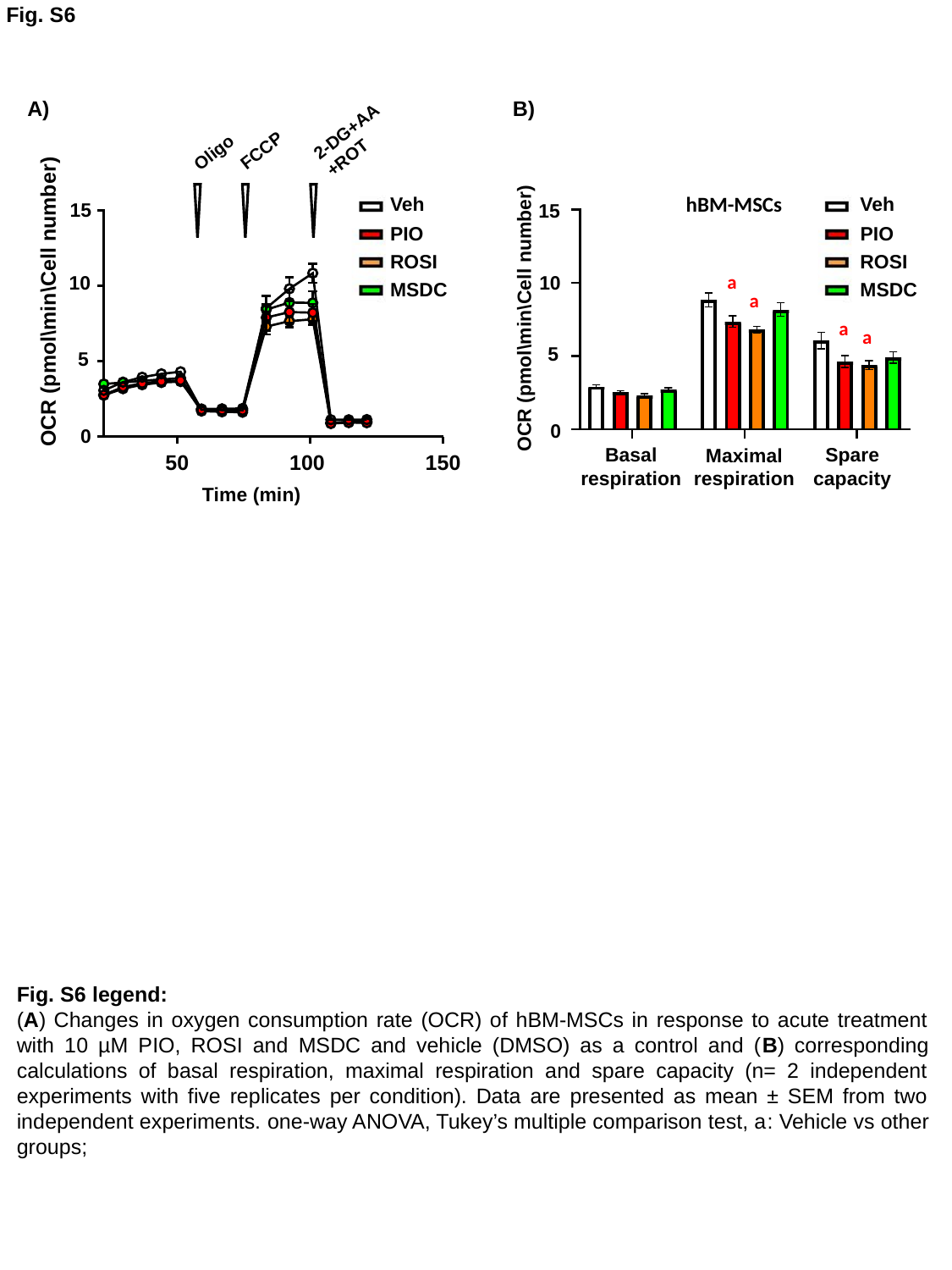

Fig. S6
2-DG+AA
+ROT
FCCP
Oligo
A)
B)
hBM-MSCs
Veh
Veh
15
15
PIO
PIO
ROSI
ROSI
a
10
10
OCR (pmol\min\Cell number)
MSDC
MSDC
OCR (pmol\min\Cell number)
a
a
a
5
5
0
0
Basal respiration
Spare capacity
Maximal respiration
50
100
150
Time (min)
Fig. S6 legend:
(A) Changes in oxygen consumption rate (OCR) of hBM-MSCs in response to acute treatment with 10 µM PIO, ROSI and MSDC and vehicle (DMSO) as a control and (B) corresponding calculations of basal respiration, maximal respiration and spare capacity (n= 2 independent experiments with five replicates per condition). Data are presented as mean ± SEM from two independent experiments. one-way ANOVA, Tukey’s multiple comparison test, a: Vehicle vs other groups;

## Slide 10
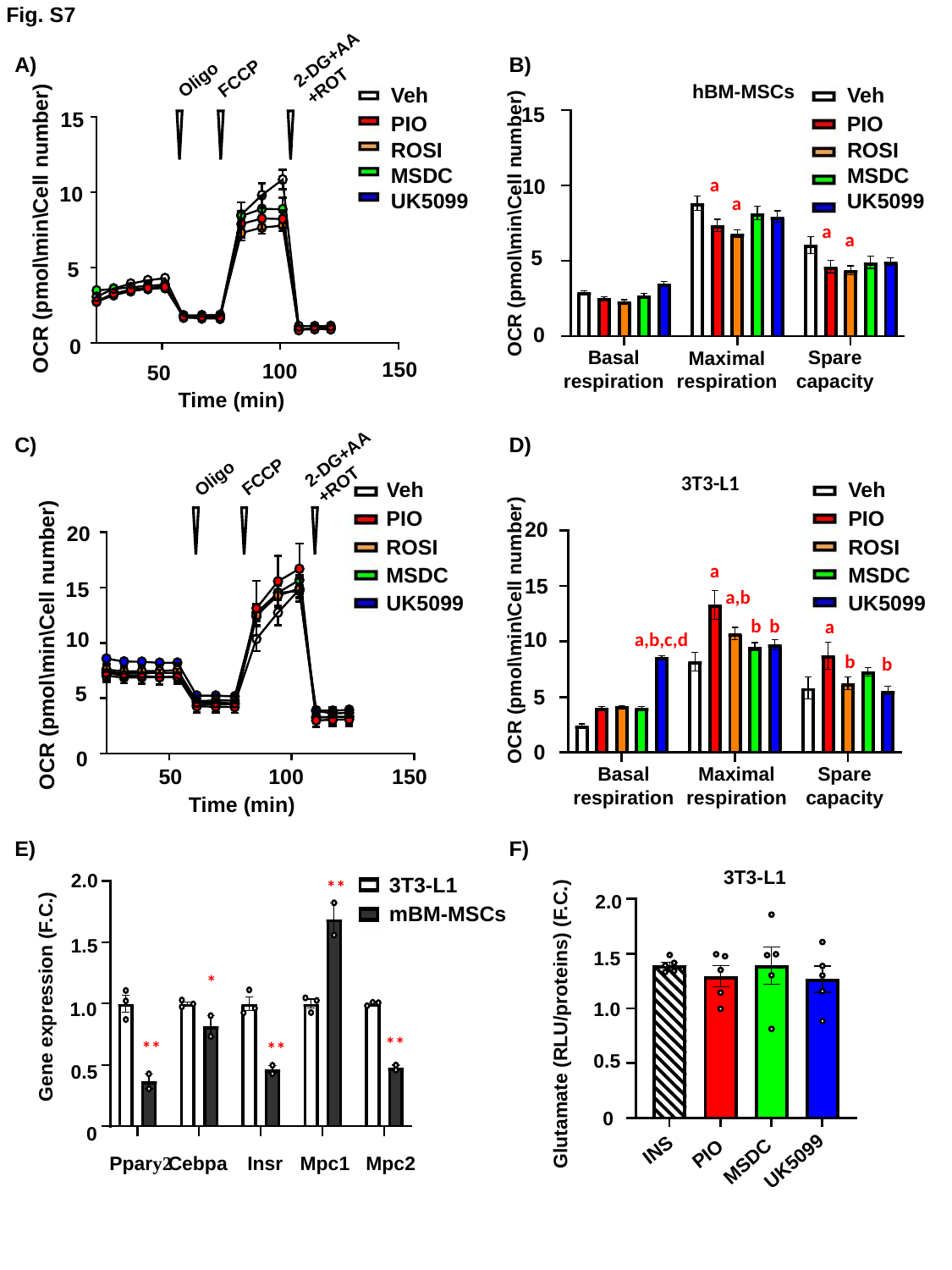

Fig. S7
2-DG+AA
+ROT
FCCP
Oligo
15
10
OCR (pmol\min\Cell number)
5
0
150
100
50
Time (min)
A)
B)
hBM-MSCs
15
a
10
a
a
a
5
0
Basal respiration
Spare capacity
Maximal respiration
Veh
Veh
PIO
PIO
ROSI
ROSI
MSDC
MSDC
OCR (pmol\min\Cell number)
UK5099
UK5099
C)
D)
FCCP
Oligo
15
OCR (pmol\min\Cell number)
10
5
0
50
100
150
Time (min)
20
2-DG+AA
+ROT
3T3-L1
Veh
Veh
PIO
PIO
20
ROSI
ROSI
a
MSDC
MSDC
15
a,b
UK5099
UK5099
OCR (pmol\min\Cell number)
b
b
a
10
a,b,c,d
b
b
5
0
Basal respiration
Spare capacity
Maximal respiration
E)
F)
3T3-L1
2.0
3T3-L1
**
2.0
mBM-MSCs
1.5
1.5
Gene expression (F.C.)
*
1.0
1.0
Glutamate (RLU/proteins) (F.C.)
**
**
**
0.5
0.5
0
 0
INS
PIO
UK5099
MSDC
Ppary2
Cebpa
Insr
Mpc1
Mpc2

## Slide 11
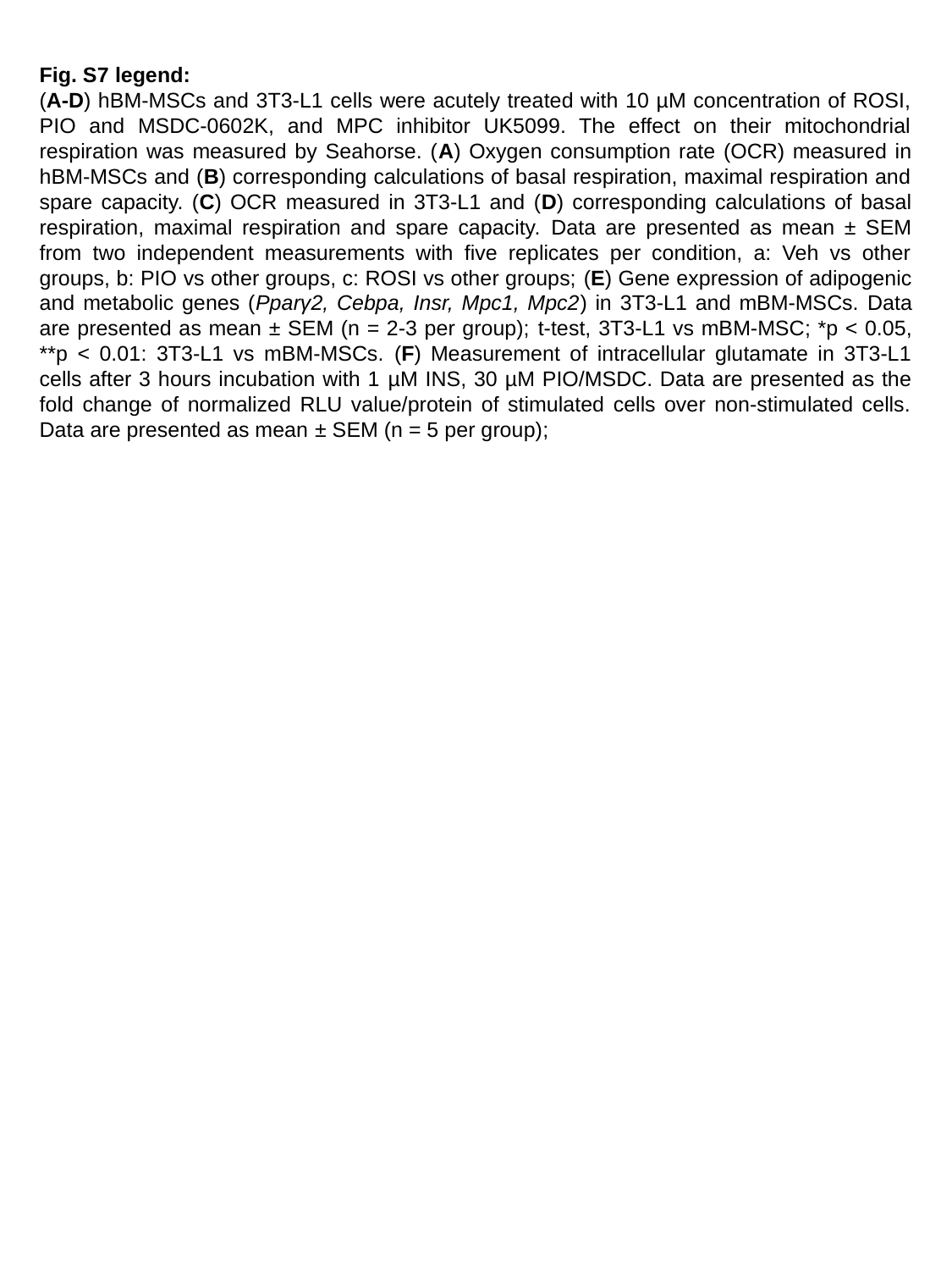

Fig. S7 legend:
(A-D) hBM-MSCs and 3T3-L1 cells were acutely treated with 10 µM concentration of ROSI, PIO and MSDC-0602K, and MPC inhibitor UK5099. The effect on their mitochondrial respiration was measured by Seahorse. (A) Oxygen consumption rate (OCR) measured in hBM-MSCs and (B) corresponding calculations of basal respiration, maximal respiration and spare capacity. (C) OCR measured in 3T3-L1 and (D) corresponding calculations of basal respiration, maximal respiration and spare capacity. Data are presented as mean ± SEM from two independent measurements with five replicates per condition, a: Veh vs other groups, b: PIO vs other groups, c: ROSI vs other groups; (E) Gene expression of adipogenic and metabolic genes (Pparγ2, Cebpa, Insr, Mpc1, Mpc2) in 3T3-L1 and mBM-MSCs. Data are presented as mean ± SEM (n = 2-3 per group); t-test, 3T3-L1 vs mBM-MSC; *p < 0.05, **p < 0.01: 3T3-L1 vs mBM-MSCs. (F) Measurement of intracellular glutamate in 3T3-L1 cells after 3 hours incubation with 1 µM INS, 30 µM PIO/MSDC. Data are presented as the fold change of normalized RLU value/protein of stimulated cells over non-stimulated cells. Data are presented as mean ± SEM (n = 5 per group);
