# Supplementary material for: Novel thiazolidinedione analog reduces a negative impact on bone and mesenchymal stem cell properties in obese mice compared to classical thiazolidinediones
Source: Mol Metab. 2022 Sep 11;65:101598. doi: 10.1016/j.molmet.2022.101598 (PMC9508355; doi:10.1016/j.molmet.2022.101598)
Supplement: Multimedia component 2 [file mmc2.docx]

**SUPPLEMENTARY MATERIAL**

Supplemental Material and Methods

Supplemental Tables: Table S1-S4

Supplemental Figures with figure legends: Fig. S1-S7

**Supplemental Material and Methods**

**Bone histology**

Tibias were collected after the dissection and fixed for 48 h in 10% formalin. After formalin fixation, bones were demineralized by EDTA 12% solution for 14 days. Tissues were embedded in paraffin and sections were used for haematoxylin eosin staining as previously published [1].

**Isolation of mRNA and quantitative RT-PCR**

Total RNA was isolated using TRI Reagent (Merck, Darmstadt, Germany) and RNA concentration was measured using Nanodrop spectrometer. cDNA synthesis was performed from 1 µg of total RNA using High-Capacity cDNA Reverse Transcription Kit (Thermo Fisher Scientific, Waltham, MA, USA) according to the manufacturer protocol. Quantitative real-time PCR was performed using Light Cycler® 480 SYBR Green I Master (Roche, Basel, Switzerland) with specific primers (GeneriBiotech, Hradec Králové, Czech Republic) listed in **Table S1** and **Table S2**. RT-PCR data were normalized to the housekeeping gene expression (36B4 for mouse, β-ACTIN for human).

**Western blot**

Protein lysates from the cells were prepared using M2 lysis buffer. Protein concentration was measured using BCA assay (Thermo Fisher Scientific, Waltham, MA, USA). Proteins with a final loading concentration of 15 µg/mL were separated in sodium dodecyl sulphate polyacrylamide gels and transferred onto PVDF (polyvinylidene difluoride) membrane (Imobilon-P) by semi-dry electroblotting. Due to higher number of experimental groups, internal control was loaded into each membrane with loading concentration similar to concentration of the samples. After blotting, membranes were washed for 5 minutes in TBS (150 mM Tris-HCl, 10 mM NaCl; pH 7,4) and blocked in 5% (*w/v*) fat-free dry milk diluted in TBS-T (TBS with 1% (*v/v*) detergent Tween-20) for 1 hour. After blocking, the membranes were washed 5 x 5 minutes in TBS-T. For immunodetection, the membranes were incubated with primary antibody (diluted in 5% milk) overnight in 4 °C. Next day, membranes were washed 5 x 5 minutes. in TBS-T and then incubated with corresponding HRP-conjugated secondary antibody for 1 hour at RT. The list of WB antibodies (Cell Signaling, Danvers, MA, USA) is presented in **Table S3**. Protein detection was performed using ECL Clarity Max detection substrate (Bio-Rad) measured by ChemiDoc imaging system (Bio-Rad, Hercules, CA, USA) and signals were calculated by Image Lab software (Bio-Rad, Hercules, CA, USA). Densitometry analysis was normalized to signals from positive control protein lysates present in each membrane.

**Lipidomics and metabolomics**

Global lipidomic and metabolomic profiling of BM, bone powder (BP), and plasma samples was conducted using a combined untargeted and targeted workflow for the lipidome, metabolome, and exposome analysis (LIMeX) [2-4] with some modifications. Extraction was carried out using a biphasic solvent system of cold methanol, methyl *tert*-butyl ether (MTBE), and 10% methanol. Four different liquid chromatography-mass spectrometry (LC-MS) platforms were used for profiling: (i) lipidomics of complex lipids using reversed-phase liquid chromatography with mass spectrometry (RPLC-MS) in positive ion mode, (ii) lipidomics of complex lipids in RPLC-MS in negative ion mode, (iii) metabolomics of polar metabolites using hydrophilic interaction chromatography with mass spectrometry (HILIC-MS) in positive ion mode, and (iv) metabolomics of polar metabolites using RPLC-MS in negative ion mode.

**Sample extraction for metabolomic and lipidomic analyses**

BM and BP samples (20–25 mg) were homogenized with 275 µL methanol containing internal standards (PE 17:0/17:0, PG 17:0/17:0, LPC 17:1, Sphingosine d17:1, Cer d18:1/17:0, SM d18:1/17:0, PC 15:0/18:1-*d*_7_, cholesterol-*d*_7_, TG 17:0/17:1/17:0-*d*_5_, DG 12:0/12:0/0:0, DG 18:1/2:0/0:0, LPE 17:1, oleic acid-*d*_9_, PI 15:0/18:1-*d*_7_, MG 17:0/0:0/0:0, PS 17:0/17:0, HexCer d18:1/17:0, DG 18:1/0:0/18:1-*d*_5_, TG 20:0/20:1/20:0-*d*_5_, LPG 17:1, LPS 17:1, cardiolipin 16:0/16:0/16:0/16:0) and 275 µL 10% methanol containing internal standards (caffeine-*d*_9_, acetylcholine-*d*_4_, creatinine-*d*_3_, choline-*d*_9_, TMAO-*d*_9_, *N*-methylnicotinamide-*d*_4_, betaine-*d*_9_, butyrobetaine-*d*_9_, creatine-*d*_3_, cotinine-*d*_3_, glucose-*d*_7_, succinic acid-*d*_4_, metformin-*d*_6_) for 1.5 min using a grinder (MM400, Retsch, Germany). Then, 1 mL of MTBE with internal standard (CE 22:1) was added, the tubes were shaken for 1 min and centrifuge at 16,000 rpm for 5 min.

Plasma samples (25 µL) were mixed with 765 μL of cold methanol/MTBE mixture (165 µL + 600 µL, respectively) containing the same mixtures of internal standards as before and shaken for 30 s. Then, 165 µL of 10% MeOH with deuterated polar metabolite internal standards was added, shaken for 30 s, and centrifuged at 16,000 rpm for 5 min.

For lipidomic profiling, 100 µL of upper organic phase was collected, evaporated and resuspended using 100 µL methanol with internal standard (12-[[(cyclohexylamino)carbonyl]amino]-dodecanoic acid, CUDA), shaken for 30 s, centrifuged at 16,000 rpm for 5 min and used for LC-MS analysis.

For metabolomic profiling, 70 µL of bottom aqueous phase was collected, evaporated, resuspended in 70 µL of an acetonitrile/water (4:1, *v*/*v*) mixture with internal standards (CUDA and Val-Tyr-Val), shaken for 30 s, centrifuged at 16,000 rpm for 5 min and analyzed using HILIC metabolomics platform. Another 70 µL aliquote of bottom aqueous phase was mixed with 210 µL of an isopropanol/acetonitrile (1:1, *v*/*v*) mixture, shaken for 30 s, centrifuged at 16,000 rpm for 5 min, and the supernatant was evaporated, resuspended in 5% methanol/0.2% formic acid with internal standards (CUDA and Val-Tyr-Val), shaken for 30 s, centrifuged at 16,000 rpm for 5 min and analyzed using RPLC metabolomics platform.

**LC-MS-based lipidomics**

The LC-MS systems consisted of a Vanquish UHPLC System (Thermo Fisher Scientific, Waltham, MA, USA) coupled to a Q Exactive Plus mass spectrometer (Thermo Fisher Scientific, Waltham, MA, USA).

Lipids were separated on an Acquity UPLC BEH C18 column (50 × 2.1 mm; 1.7 μm) coupled to an Acquity UPLC BEH C18 VanGuard pre-column (5 × 2.1 mm; 1.7 μm) (Waters, Milford, MA, USA). The column was maintained at 65°C at a flow-rate of 0.6 mL/min. For LC–ESI(+)-MS analysis, the mobile phase consisted of (A) 60:40 (*v*/*v*) acetonitrile:water with ammonium formate (10 mM) and formic acid (0.1%) and (B) 90:10:0.1 (*v*/*v*/*v*) isopropanol:acetonitrile:water with ammonium formate (10 mM) and formic acid (0.1%). For LC–ESI(−)-MS analysis, the composition of the solvent mixtures were the same with the exception of the addition of ammonium acetate (10 mM) and acetic acid (0.1%) as mobile-phase modifiers. Separation was conducted under the following gradient for LC–ESI(+)-MS: 0 min 15% (B); 0–1 min 30% (B); 1–1.3 min from 30% to 48% (B); 1.3–5.5 min from 48% to 82% (B); 5.5–5.8 min from 82% to 99% (B); 5.8–6 min 99% (B); 6–6.1 min from 99% to 15% (B); 6.1–7.5 min 15% (B). For LC–ESI(−)-MS, the following gradient was used: 0 min 15% (B); 0–1 min 30% (B); 1–1.3 min from 30% to 48% (B); 1.3–4.8 min from 48% to 76% (B); 4.8–4.9 min from 76% to 99% (B); 4.9–5.3 min 99% (B); 5.3–5.4 min from 99% to 15% (B); 5.4–6.8 min 15% (B). A sample volume of 0.3 µL, 1.5 µL, and 1.5 µL was used for bone marrow, bone powder, and plasma extracts, respectively, in ESI(+). A sample volume of 5 µL for all matrices was used in ESI(–). Sample temperature was maintained at 4°C.

The ESI source and MS parameters were: sheath gas pressure, 60 arbitrary units; aux gas flow, 25 arbitrary units; sweep gas flow, 2 arbitrary units; capillary temperature, 300°C; aux gas heater temperature, 370°C; MS1 mass range, *m*/*z* 200–1700; MS1 resolving power, 35,000 FWHM (*m/z* 200); number of data-dependent scans per cycle, 3; MS/MS resolving power, 17,500 FWHM (*m/z* 200). For ESI(+), a spray voltage of 3.6 kV and normalized collision energy of 20% was used while for ESI(−) a spray voltage of −3.0 kV and normalized collision energy of 10, 20 and 30% were set-up.

**LC-MS-based metabolomics**

Polar metabolites were separated on an Acquity UPLC BEH Amide column (50 × 2.1 mm; 1.7 μm) coupled to an Acquity UPLC BEH Amide VanGuard pre-column (5 × 2.1 mm; 1.7 μm) (Waters, Milford, MA, USA). The column was maintained at 45°C at a flow-rate of 0.4 mL/min. The mobile phase consisted of (A) water with ammonium formate (10 mM) and formic acid (0.125%) and (B) acetonitrile:water (95/5) with ammonium formate (10 mM) and formic acid (0.125%). Separation was conducted under the following gradient: 0 min 100% (B); 0–1 min 100% (B); 1–3.9 min from 100% to 70% (B); 3.9–5.1 min from 70% to 30% (B); 5.1–6.4 min from 30% to 100%(B); 6.4–8.0 min 100% (B). A sample volume of 0.5 µL, 0.5 µL, and 1.5 µL was used for bone marrow, bone powder, and plasma extracts, respectively, in ESI(+). Sample temperature was maintained at 4°C.

Polar metabolites were also separated on an Acquity UPLC HSS T3 column (50 × 2.1 mm; 1.8 μm) coupled to an Acquity UPLC HSS T3 VanGuard pre-column (5 × 2.1 mm; 1.8 μm) (Waters, Milford, MA, USA). The column was maintained at 45°C using a ramped flow-rate. The mobile phase consisted of (A) water with formic acid (0.2%) and (B) methanol with formic acid (0.1%). Separation was conducted under the following gradient: 0 min 1% (B) 0.3 mL/min; 0–0.5 min 1% (B) 0.3 mL/min; 0.5–2 min from 1% to 60% (B) 0.3 mL/min; 2–2.3 min from 60% to 95% (B) from 0.3 mL/min to 0.5 mL/min; 2.3–3.0 min 95% (B) 0.5 mL/min; 3.0–3.1 min from 95% to 1% (B) 0.5 mL/min; 3.1–4.5 min 1% (B) 0.5 mL/min; 4.5–4.6 min 1% (B) from 0.5 mL/min to 0.3 mL/min; 4.6–5.5 min 1% (B) 0.3 mL/min. A sample volume of 5 μL was used for the injection in ESI(–). Sample temperature was maintained at 4°C.

The ESI source and MS parameters were: sheath gas pressure, 50 arbitrary units; aux gas flow, 13 arbitrary units; sweep gas flow, 3 arbitrary units; capillary temperature, 260°C; aux gas heater temperature, 425°C; MS1 mass range, *m/z* 60–900; MS1 resolving power, 35,000 FWHM (*m/z* 200); number of data-dependent scans per cycle, 3; MS/MS resolving power, 17,500 FWHM (*m/z* 200). A spray voltage of 3.6 kV and −2.5 kV for ESI(+) and ESI(–), respectively, was used. For all metabolomics platforms a normalized collision energy of 20, 30 and 40% was used.

**Quality control**

Quality control was assured by (i) randomization of the actual samples within the sequence, (ii) injection of quality control (QC) pool samples at the beginning and the end of the sequence and between each 10 actual samples, (iii) analysis of procedure blanks, (iv) serial dilution of QC sample (0, 1/16, 1/8, 1/4, 1/2, 1), (v) checking the peak shape and the intensity of spiked internal standards and the internal standard added prior to injection.

**Data processing**

LC-MS data from metabolomic and lipidomic profiling were processed through MS-DIAL v. 4.70 software. Metabolites were annotated using in-house retention time–*m*/*z* library and using MS/MS libraries available from commercial and open sources (NIST20, MassBank, MoNA). Lipids were annotated using LipidBlast in-built in MS-DIAL. Traces of pioglitazone (*m*/*z* 355.1116) and MSDC-0602K (*m*/*z* 370.0749) were detected in RPLC-MS lipidomics in ESI (–) as deprotonated molecules. Raw data were filtered using blank samples, serial dilution samples, and QC pool samples with relative standard deviation (RSD) <30%, and then normalized using locally estimated scatterplot smoothing (LOESS) approach by means of QC pool samples injected regularly between 10 actual samples followed by sample-weight and injection volume normalization. Data were exported as the detector signal intensity in arbitrary units (A.U.).

**Biochemical analyses of bone turnover markers**

Rat/Mouse TRAP EIA for the quantitative determination of the Tartrate-resistant acid phosphatase (TRAP) for bone resorption and Rat/Mouse P1NP EIA for the determination of the N-terminal propeptide of type I procollagen (P1NP) for bone formation (MyBioSource, San Diego, CA, USA) were measured in mouse serum samples.

**Cellular Reactive Oxygen Species (ROS) Detection assay**

DCFDA (2,7-dichloro-dihydro-fluoroscein diacetate) (Abcam, Cambridge, United Kingdom) was used to measure the intracellular ROS production of primary mBM-MSCs [5]. Cells were seeded to a dark, clear bottom 96-well plate at a density of 25000 cells/well to adhere overnight. The next day, culture growth media was replaced with DCFDA Solution (25 μM) and incubated for 45 minutes at 37 °C and 5 % CO_2_. DCFDA was removed and cells were loaded with 1x Buffer supplemented with 50 µM TBHP (tert-butyl hydrogen peroxide) for 1 hour at 37 °C and 5 % CO_2_. The fluorescent intensity was detected every minute for 30 minutes using a fluorescent microplate reader (Excitation ~485 nm/Emission ~535 nm). The results were expressed as % of ROS production.

**Glucose uptake assay**

The Glucose Uptake-Glo Assay (Promega, Madison, WI, USA) was used to measure glucose uptake in hBM-MSC and 3T3-L1 cells after short term treatment with insulin, TZDs, TZD analog MSDC-0602K and MPC inhibitor UK5099. Cells were seeded at the density of 10 000 cells per well in 96-well black plate with clear bottom. Following day, cells were washed with PBS and cultured with serum-free MEM alpha (Thermo Fisher Scientific, Waltham, MA, USA) medium with 0.5 % BSA overnight. Next day, media was replaced with basal DMEM media (Thermo Fisher Scientific, Waltham, MA, USA) with 0.5 % BSA supplemented with 1 µM insulin (+INS), 30 µM PIO, ROSI and MSDC-0602K and 2 µM UK5099. After 1.5-hour incubation media was replaced with 1 mM 2-deoxyglucose (2DG). The reaction was stopped after 15 minutes incubation, and samples were prepared for measurement of luminescence according to the manufacturer protocol. Luminescence signal was detected using Tecan infinity M200 reader (Tecan, Männedorf, Switzerland) and relative light units (RLU) were normalized to protein content of samples measured by BCA assay (Thermo Fisher Scientific, Waltham, MA, USA). The data are expressed as the fold change of the normalized RLU values/ protein of non-stimulated cells.

**Glutamine/Glutamate assay**

The Glutamine-Glutamate-Glo Assay (Promega, Madison, WI, USA) was used to detect glutamine and glutamate in hBM-MSC and 3T3-L1 cells after short-term treatment with 1µM insulin, 30 µM PIO, and 30 µM TZD analog MSDC-0602K. Cells were seeded at the density of 15 000 cells per well in 96-well plate. Following day, cells were washed with PBS and cultured with serum-free MEM alpha (Thermo Fisher Scientific, Waltham, MA, USA) medium with 0.5 % BSA (Merck, Darmstadt, Germany) overnight. Next day, starvation media was replaced with basal DMEM media (Thermo Fisher Scientific, Waltham, MA, USA) with 2mM glutamine, 5mM glucose supplemented with 1 µM insulin (+INS), 30 µM PIO, ROSI and MSDC-0602K and 2 µM UK5099. After 3 hours incubation, the cells were washed twice with PBS and then processed with PBS containing Inactivation solution followed by Tris solution. Cell lysates were transferred to the white 96-well plate for the measurement of glutamine/glutamate according to the manufacturer protocol. Luminescent signal was detected using Tecan infinity M200 reader (Tecan, Männedorf, Switzerland) and relative light units (RLU) were normalized to protein content of samples measured by BCA assay (Thermo Fisher Scientific, Waltham, MA, USA). The data are expressed as the fold change of the normalized RLU values/ protein of non-stimulated cells.

**References**

[1] Tencerova M, Figeac F, Ditzel N, Taipaleenmaki H, Nielsen TK, Kassem M (2018) High-Fat Diet-Induced Obesity Promotes Expansion of Bone Marrow Adipose Tissue and Impairs Skeletal Stem Cell Functions in Mice. J Bone Miner Res 33(6): 1154-1165. 10.1002/jbmr.3408

[2] Sistilli G, Kalendova V, Cajka T, et al. (2021) Krill Oil Supplementation Reduces Exacerbated Hepatic Steatosis Induced by Thermoneutral Housing in Mice with Diet-Induced Obesity. Nutrients 13(2). 10.3390/nu13020437

[3] Janovska P, Melenovsky V, Svobodova M, et al. (2020) Dysregulation of epicardial adipose tissue in cachexia due to heart failure: the role of natriuretic peptides and cardiolipin. J Cachexia Sarcopenia Muscle 11(6): 1614-1627. 10.1002/jcsm.12631

[4] Tsugawa H, Ikeda K, Takahashi M, et al. (2020) A lipidome atlas in MS-DIAL 4. Nat Biotechnol 38(10): 1159-1163. 10.1038/s41587-020-0531-2

[5] Tencerova M, Frost M, Figeac F, et al. (2019) Obesity-Associated Hypermetabolism and Accelerated Senescence of Bone Marrow Stromal Stem Cells Suggest a Potential Mechanism for Bone Fragility. Cell Rep 27(7): 2050-2062 e2056. 10.1016/j.celrep.2019.04.066

**SUPPLEMENTAL TABLES**

**Table S1**: List of mouse primers

| **Gene name** | **Gene Sequence 5’-3’** |
| --- | --- |
| ***36B4 F*** | TCCAGGCTTTGGGCATCA |
| ***36B4 R*** | CTTTATCAGCTGCACATCACTCAGA |
| ***Fsp27 F*** | ATCAGAACAGCGCAAGAAGA |
| ***Fsp27 R*** | CAGCTTGTACAGGTCGAAGG |
| ***Cd36 F*** | ATGGGCTGTGATCGGAACTG |
| ***Cd36 R*** | TTTGCCACGTCATCTGGGTTT |
| ***Alp F*** | GCCCTCTCCAAGACATATA |
| ***Alp R*** | CCATGATCACGTCGATATCC |
| ***Bmp2 F*** | GGGACCCGCTGTCTTCTAGT |
| ***Bmp2 R*** | TCAACTCAAATTCGCTGAGGAC |
| ***Col1a1 F*** | GGTGAACAGGGGTTCCTGG |
| ***Col1a1 R*** | TTCGCACCAGGTTGCCATC |
| ***Adipoq F*** | GACGTTACTACAACTGAAGAGC |
| ***Adipoq R*** | CATTCTTTTCCTGATACTGGTC |
| ***Cebpa F*** | AAGCCAAGAAGTCGGTGGA |
| ***Cebpa R*** | CAGTTCACGGCTCAGCTGTTC |
| ***Il1β F*** | GCAACTGTTCCTGAACTCAACT |
| ***Il1β R*** | ATCTTTTGGGGTCCGTCAACT |
| ***Tnfα F*** | CCCTCACACTCAGATCATCTTCT |
| ***Tnfα R*** | GCTACGACGTGGGCTACAG |
| ***Mpc1 F*** | TCATTCAGGGAGGACGACTTATC |
| ***Mpc1 R*** | TGTTTTCCCTTCAGCACGACTAC |
| ***Mpc2 F*** | CTCCCACCCTGCTGCTGTCG |
| ***Mpc2 R*** | GGCCTGCCGGGTGGTTGTA |
| ***p53 F*** | TCTTATCCGGGTGGAAGGAAA |
| ***p53 R*** | GGCGAAAAGTCTGCCTGTCTT |
| ***p16 F*** | GGGTTTTCTTGGTGAAGTTCG |
| ***p16 R*** | TTGCCCATCATCATCACCT |
| ***Sod2 F*** | CAGACCTGCCTTACGACTATGG |
| ***Sod2 R*** | CTCGGTGGCGTTGAGATTGTT |
| ***Hmox1 F*** | AGGTACACATCCAAGCCGAGA |
| ***Hmox1 R*** | CATCACCAGCTTAAAGCCTTCT |
| ***Pparγ2 F*** | GGGTCAGCTCTTGTGAATGG |
| ***Pparγ2 R*** | CTGATGCACTGCCTATGAGC |
| ***Pdk4 F*** | GGCTTGCCAATTTCTCGTCTCTA |
| ***Pdk4 R*** | TTCGCCAGGTTCTTCGGTTCC |
| ***Pc F*** | CCCCTGGATAGCCTTAATACTCGT |
| ***Pc R*** | TGGCCCTTCACATCCTTCAAA |
| ***Oc F*** | TGCGCTCTGTCTCTCTGACC |
| ***Oc R*** | CTGTGACATCCATACTTGCAGG |
| ***p21 F*** | CCTGGTGATGTCCGACCTG |
| ***p21 R*** | CCATGAGCGCATCGCAATC |
| ***Trap F*** | CAGCTCCCTAGAAGATGGATTCAT |
| ***Trap R*** | GTCAGGAGTGGGAGCCATATG |
| ***Rankl F*** | AGCCGAGACTACGGCAAGTA |
| ***Rankl R*** | AAAGTACAGGAACAGAGCGATG |
| ***Opg F*** | CCTTGCCCTGACCACTCTTAT |
| ***Opg R*** | CACACACTCGGTTGTGGGT |
| ***Ctsk F*** | AGGCAGCTAAATGCAGAGGGTACA |
| ***Ctsk R*** | AGCTTGCATCGATGGACACAGAGA |
| ***RelA F*** | ACTGCCGGGATGGCTACTAT |
| ***RelA R*** | TCTGGATTCGCTGGCTAATGG |
| ***Irs1 F*** | TCTACACCCGAGACGAACACT |
| ***Irs1 R*** | TGGGCCTTTGCCCGATTATG |
| ***Irs2 F*** | CTGCGTCCTCTCCCAAAGTG |
| ***Irs2 R*** | GGGGTCATGGGCATGTAGC |
| ***Insr F*** | ATGGGCTTCGGGAGAGGAT |
| ***Insr R*** | CTTCGGGTCTGGTCTTGAACA |
| ***Slc1a5 F*** | CAGGCAGGCTGACACTGGAT |
| ***Slc1a5 R*** | TGGAGATGAAAGACGTCCGC |
| ***Slc2a4 /Glut4 F*** | CTCATGGGCCTAGCCAATGC |
| ***Slc2a4/ Glut4 R*** | CCCTGATGTTAGCCCTGAGTA |
| ***Gls F*** | AGGGTGAAGTCGGTGATAAAC |
| ***Gls R*** | GGGCTGTTCTGGAGTCATAAT |
| ***Gls2 F*** | CAACTTCAATGTGCCCTTCAG |
| ***Gls2 R*** | CTGCATATAGTGGAGATGTCTCG |
| ***Ctnnb1 F*** | CCCAGTCCTTCACGCAAGAG |
| ***Ctnnb1 R*** | CATCTAGCGTCTCAGGGAACA |
| ***Dlx5 F*** | CACCACCCGTCTCAGGAATC |
| ***Dlx5 F*** | GCTTTGCCATAAGAAGCAGAGG |
| ***Lrp5 F*** | CCGAGGGAGCCTTTCTACTC |
| ***Lrp5 R*** | CCCTGTCTTGCACGTCTTG |
| ***Msx2 F*** | CTAAAGGCGGTGACTTGTTTTCG |
| ***Msx2 R*** | CGGCTTCTTGTCGGACATGAG |
| ***Vegfa F*** | GTACCTCCACCATGCCAAGTG |
| ***Vegfa R*** | TGGGACTTCTGCTCTCCTTCTG |
| ***Vcam F*** | GGCTCCAGACATTTACCCAGTT |
| ***Vcam R*** | CATGAGCTGGTCACCCTTGAA |
| ***Fas F*** | CTGCACCCTGACCCAGAATAC |
| ***Fas R*** | ACAGCCAGGAGAATCGCAGTA |
| ***Fasgl F*** | CAGTCCACCCCCTGAAAAAAA |
| ***Fasgl R*** | CCTTGAGTTGGACTTGCCTGTT |
| ***Il10 F*** | CTGGACAACATACTGCTAACCG |
| ***Il10 R*** | GGGCATCACTTCTACCAGGTAA |
| ***Il1rn F*** | GCTCATTGCTGGGTACTTACAA |
| ***Il1rn R*** | CCAGACTTGGCACAAGACAGG |

**Table S2**. List of human primers

| **Gene name** | **Gene Sequence 5’-3’** |
| --- | --- |
| **β-ACTIN F** | ATTGGCAATGAGCGGTTCCG |
| **β-ACTIN R** | AGGGCAGTGATCTCCTTCTG |
| **ALPL F** | ACGTGGCTAAGAATGTCATC |
| **ALPL R** | CTGGTAGGCGATGTCCTTA |
| **MPC 1 F** | TCATGAGTACGCACTTCTGGGGC |
| **MPC 1 R** | GCCAGTTCCGAGGCTGTACCT |
| **MPC 2 F** | CTCTAGGCGGCGACCTCAGC |
| **MPC 2 R** | GGAAAAGGTCCCTCGGGCTGG |
| **PPARG F** | CTCCTATTGACCCAGAAAGCGA |
| **PPARG R** | TGCCATGAGGGAGTTGGAAG |
| **CEBPA F** | AACCTTGTGCCTTGGAAATG |
| **CEBPA R** | CTGTAGCCTCGGGAAGGAG |
| **INSR F** | TACTTGGCCACTATCGACTGG |
| **INSR R** | GCCGTGTGACTTACAGATGGT |
| **IRS1 F** | CCCAGGACCCGCATTCAAA |
| **IRS1 R** | GGCGGTAGATACCAATCAGGT |
| **ADIPOQ F** | GGGCCCCAGGCCGTGATGGCA |
| **ADIPOQ R** | TCGGGGACCTTCAGCCCCGGGTA |
| **SLC1A5 F** | TCATGTGGTACGCCCCTGT |
| **SLC1A5 R** | GCGGGCAAAGAGTAAACCCA |
| **GLS F** | AGGGTCTGTTACCTAGCTTGG |
| **GLS R** | ACGTTCGCAATCCTGTAGATTT |
| **GSS F** | GGGAGCCTCTTGCAGGATAAA |
| **GSS R** | GAATGGGGCATAGCTCACCAC |

**Table S3**. List of primary and secondary antibodies used for western blot

| **Primary antibodies** | **Company** | **Dilution** |
| --- | --- | --- |
| Phospho-Akt Ser473_ Rabbit | Cell Signaling | 1:1000 |
| Phospho-Akt Thr308_Rabbit | Cell Signaling | 1:1000 |
| Total AKT_Rabbit | Cell Signaling | 1:1000 |
| β-actin_Rabbit | Cell Signaling | 1:1000 |
| **Secondary HRP-conjugated antibodies** |  |  |
| Anti-rabbit IgG, HRP-linked Antibody | Cell Signaling | 1:5000 |

**Table S4.** List of plasma and BM unique metabolites.

| **Plasma unique metabolites** | **BM unique metabolites** |
| --- | --- |
| CE 16:0; [M+NH4]+ | CAR 16:0-OH; [M]+ |
| CE 18:1; [M+NH4]+ | CAR 18:2 (2); [M+H]+ |
| CE 18:3; [M+NH4]+ | CL 72:7; CL 18:1_18:2_18:2_18:2; [M-H]- |
| CE 20:3; [M+NH4]+ | CL 72:8; CL 18:2_18:2_18:2_18:2; [M-H]- |
| CE 20:5; [M+NH4]+ | Cer 36:0;2O; Cer 18:0;2O/18:0; [M+CH3COO]- |
| CE 22:5; [M+NH4]+ | Cer 42:2;2O (1); Cer 18:0;2O/24:2; [M+CH3COO]- |
| CE 22:6; [M+NH4]+ | Cer 34:2;2O; Cer 18:2;2O/16:0; [M+CH3COO]- |
| FA 25:1; [M-H]- | Cer 36:2;2O; Cer 18:2;2O/18:0; [M+CH3COO]- |
| LPC 20:5/0:0 (1); [M+H]+ | DG 32:1; DG 16:0_16:1; [M+NH4]+ |
| LPC 22:5/0:0 (1); [M+H]+ | DG 38:4 (2); DG 16:0_22:4; [M+NH4]+ |
| LPC 22:5/0:0 (2); [M+H]+ | DG 40:5; DG 18:1_22:4; [M+NH4]+ |
| PC 39:6; [M+H]+ | FA 21:2; [M-H]- |
| PC 39:7; [M+H]+ | FA 24:3; [M-H]- |
| PC 42:6; [M+H]+ | FA 26:2; [M-H]- |
| SM 42:5;2O; [M+CH3COO]- | FA 26:4; [M-H]- |
| TG 58:10 (2); TG 16:0_20:4_22:6; [M+NH4]+ | FA 26:5; [M-H]- |
| TG O-58:1; TG O-20:0_18:0_20:1; [M+NH4]+ | FA 26:6; [M-H]- |
| 4-Hydroxyhippuric acid; [M-H]- | HexCer 36:1;2O; [M+CH3COO]- |
| 4-Pyridoxic acid; [M-H]- | LPG 16:0; [M-H]- |
| Cholic acid; [M+NH4]+ | LPG 18:1; [M-H]- |
| H-Pro-Hyp-OH; [M+H]+ | LPI 18:1; [M-H]- |
| Suberylglycine; [M-H]- | LPS 18:0; [M-H]- |
| Taurocholic acid; [M+H]+ | LPS 20:4; [M-H]- |
|  | LPS 22:6; [M-H]- |
|  | PE 32:0; PE 16:0_16:0; [M-H]- |
|  | PE 32:1; PE 16:0_16:1; [M-H]- |
|  | PE 38:3 (2); PE 18:0_20:3; [M-H]- |
|  | PE 40:7 (1); PE 18:2_22:5; [M-H]- |
|  | PE 40:9; PE 18:3_22:6; [M-H]- |
|  | PE 42:9; PE 20:3_22:6; [M-H]- |
|  | PE 42:10; PE 20:4_22:6; [M-H]- |
|  | PE 44:10; PE 22:4_22:6; [M-H]- |
|  | PE O-32:0; PE O-16:0_16:0; [M-H]- |
|  | PE O-36:6; PE O-16:1_20:5; [M-H]- |
|  | PE O-40:7 (1); PE O-20:3_20:4; [M-H]- |
|  | PE O-40:7 (2); PE O-18:2_22:5; [M-H]- |
|  | PE O-42:9; PE O-20:3_22:6; [M-H]- |
|  | PE 44:8;2O; PE 22:5_22:3;2O; [M-H]- |
|  | PE 44:10;2O; PE 22:6_22:4;2O; [M-H]- |
|  | PEtOH 34:1; PEtOH 16:0_18:1; [M-H]- |
|  | PEtOH 38:6; PEtOH 16:0_22:6; [M-H]- |
|  | PEtOH 40:6; PEtOH 18:0_22:6; [M-H]- |
|  | PG 36:1; PG 18:0_18:1; [M-H]- |
|  | PG 36:4 (2); PG 18:2_18:2; [M-H]- |
|  | PG 36:4 (3); PG 16:0_20:4; [M-H]- |
|  | PG 38:4; PG 18:0_20:4; [M-H]- |
|  | PG 38:5 (1); PG 18:1_20:4; [M-H]- |
|  | PG 38:5 (2); PG 18:1_20:4; [M-H]- |
|  | PG 38:6 (2); PG 16:0_22:6; [M-H]- |
|  | PG 40:6; PG 18:1_22:5; [M-H]- |
|  | PG 40:7; PG 18:1_22:6; [M-H]- |
|  | PG 40:8; PG 18:2_22:6; [M-H]- |
|  | PG 42:10; PG 20:4_22:6; [M-H]- |
|  | PG 44:11; PG 22:5_22:6; [M-H]- |
|  | PG 44:12; PG 22:6_22:6; [M-H]- |
|  | PI 40:5 (2); PI 18:0_22:5; [M-H]- |
|  | PI O-38:6; PI O-16:0_22:6; [M-H]- |
|  | PS 34:1; PS 16:0_18:1; [M-H]- |
|  | PS 36:1; PS 18:0_18:1; [M-H]- |
|  | PS 36:2; PS 18:0_18:2; [M-H]- |
|  | PS 36:3; PS 18:1_18:2; [M-H]- |
|  | PS 36:4; PS 16:0_20:4; [M-H]- |
|  | PS 38:3; PS 18:0_20:3; [M-H]- |
|  | PS 38:5; PS 18:1_20:4; [M-H]- |
|  | PS 38:6; PS 16:0_22:6; [M-H]- |
|  | PS 40:5 (2); PS 18:0_22:5; [M-H]- |
|  | PS 40:8; PS 20:4_20:4; [M-H]- |
|  | SM 42:0;2O; [M+CH3COO]- |
|  | SM 44:2;2O; [M+CH3COO]- |
|  | TG 50:7; TG 12:0_18:2_20:5; [M+NH4]+ |
|  | TG 57:7; TG 18:1_18:1_21:5; [M+NH4]+ |
|  | TG 60:12 (2); TG 16:0_22:6_22:6; [M+NH4]+ |
|  | TG O-56:7; TG O-16:0_18:1_22:6; [M+NH4]+ |
|  | 12-HETE; [M-H]- |
|  | 12-HHTrE; [M-H]- |
|  | 5-trans-Prostaglandin D2; [M-H]- |
|  | 6-trans-12-epi-Leukotriene B4; [M-H]- |
|  | Ala-Lys; [M+H]+ |
|  | Arg-Ala; [M+H]+ |
|  | Arg-Val; [M+H]+ |
|  | Asp-Leu; [M-H]- |
|  | Cytidine 3'-monophosphate; [M-H]- |
|  | Cytidine 5'-diphosphocholine; [M+H]+ |
|  | Gly-Gln; [M+H]+ |
|  | Gly-His; [M+H]+ |
|  | Guanosine 5'-monophosphate; [M-H]- |
|  | Guanosine; [M+H]+ |
|  | His-Ala; [M+H]+ |
|  | His-Asn; [M+H]+ |
|  | His-Gln; [M+H]+ |
|  | His-Gly; [M+H]+ |
|  | His-Leu; [M+H]+ |
|  | His-Ser; [M+H]+ |
|  | His-Thr; [M+H]+ |
|  | His-Tyr; [M+H]+ |
|  | His-Val; [M+H]+ |
|  | Leu-Ala; [M-H]- |
|  | Lys-Ile; [M+H]+ |
|  | Lys-Phe; [M+H]+ |
|  | Phe-Gly; [M-H]- |
|  | Prostaglandin F2-beta-; [M-H]- |
|  | Ribose 1-phosphate; [M-H]- |
|  | Thr-Lys; [M+H]+ |
|  | Thr-Tyr; [M-H]- |
|  | Uridine 5'-diphosphoacetylglucosamine; [M+H]+ |
|  | Val-Arg; [M+H]+ |
|  | Val-Gly; [M-H]- |
|  | Val-Leu; [M-H]- |
|  | Val-Val; [M-H]- |
|  | Xanthine; [M+H]+ |
